# Transcriptional Complexity and Distinct Expression Patterns of *auts2* Paralogs in *Danio rerio*

**DOI:** 10.1534/g3.117.042622

**Published:** 2017-06-16

**Authors:** Igor Kondrychyn, Lena Robra, Vatsala Thirumalai

**Affiliations:** National Centre for Biological Sciences, Tata Institute of Fundamental Research, Bangalore, Karnataka 560065, India

**Keywords:** alternative promoters, alternative splicing, auts2, fbrsl1, fbrs

## Abstract

Several genes that have been implicated in autism spectrum disorders (ASDs) have multiple transcripts. Therefore, comprehensive transcript annotation is critical for determining the respective gene function. The autism susceptibility candidate 2 (*AUTS2*) gene is associated with various neurological disorders, including autism and brain malformation. AUTS2 is important for activation of transcription of neural specific genes, neuronal migration, and neurite outgrowth. Here, we present evidence for significant transcriptional complexity in the *auts2* gene locus in the zebrafish genome, as well as in genomic loci of *auts2* paralogous genes *fbrsl1* and *fbrs*. Several genes that have been implicated in ASDs are large and have multiple transcripts. Neurons are especially enriched with longer transcripts compared to nonneural cell types. The human autism susceptibility candidate 2 (*AUTS2*) gene is ∼1.2 Mb long and is implicated in a number of neurological disorders including autism, intellectual disability, addiction, and developmental delay. Recent studies show AUTS2 to be important for activation of transcription of neural specific genes, neuronal migration, and neurite outgrowth. However, much remains to be understood regarding the transcriptional complexity and the functional roles of AUTS2 in neurodevelopment. Zebrafish provide an excellent model system for studying both these questions. We undertook genomic identification and characterization of *auts2* and its paralogous genes in zebrafish. There are four *auts2* family genes in zebrafish: *auts2a*, *auts2b*, *fbrsl1*, and *fbrs*. The absence of complete annotation of their structures hampers functional studies. We present evidence for transcriptional complexity of these four genes mediated by alternative splicing and alternative promoter usage. Furthermore, the expression of the various paralogs is tightly regulated both spatially and developmentally. Our findings suggest that auts2 paralogs serve distinct functions in the development and functioning of target tissues.

The *AUTS2* gene was originally identified in monozygotic twins concordant for autism who shared a balanced translocation from chromosome 7 to 20 (Sultana *et al.* 2002). Since that initial discovery, various structural variants disrupting the *AUTS2* locus have been identified in unrelated individuals with ASDs as well as other neuropathological conditions, including epilepsy and brain malformations [reviewed in Oksenberg and Ahituv (2013)]. Although, disruption of *AUTS2* is most often reported to be associated with neurological phenotypes, copy number variations at the *AUTS2* locus have also been observed in unaffected individuals (Redon *et al.* 2006; Bakkaloglu *et al.* 2008), indicating that structural polymorphisms are tolerated in some cases. In addition, a noncoding single nucleotide polymorphism (SNP) within *AUTS2* has been associated with schizophrenia, alcohol consumption, heroin addiction, and suicidal behavior (Chen *et al.* 2013; Chojnicka *et al.* 2013; Coon *et al.* 2013; Dang *et al.* 2014; Kapoor *et al.* 2013; McCarthy *et al.* 2014; Schumann *et al.* 2011; Zhang *et al.* 2014). *AUTS2* has also been implicated as an important gene in human-specific evolution (Prabhakar *et al.* 2006; Green *et al.* 2010).

AUTS2 is a highly conserved protein, primarily expressed in the brain in various neuronal cell types including glutamatergic, GABAergic, and dopaminergic neurons, as well as in regions implicated in autism neuropathology, such as the cerebral cortex and cerebellum (Sultana *et al.* 2002; Kalscheuer *et al.* 2007; Bedogni *et al.* 2010; Wagner *et al.* 2013). Although several features, such as putative nuclear localization sequences (NLS), two proline-rich regions (PR1 and PR2), the PY motif (PPPY), and histidine repeats, can be predicted in the human AUTS2 protein (Sultana *et al.* 2002; Oksenberg and Ahituv 2013), it does not contain any regions of homology to other proteins, except a region called the Auts2 family domain, with homology to the fibrosin (FBRS) and fibrosin-like 1 (FBRSL1) proteins. *AUTS2*, *FBRSL1*, and *FBRS* genes thus form a paralog group. Paralogs are known to be created by a duplication event within a genome and may evolve new functions. The functional roles of Auts2 family proteins and whether they are functionally diversified are not yet entirely clear.

Recently, proteomic analysis revealed that AUTS2, FBRS, and FBRSL1 are associated with the same subset of Polycomb Repressive Complex 1 (PRC1) (Gao *et al.* 2012) and, in particular, AUTS2 renders PRC1 capable of transcription activation (Gao *et al.* 2014). Though initially thought to be a nuclear protein (Bedogni *et al.* 2010), AUTS2 was shown to be also present in the cytoplasm, where it regulates Rho family GTPases to control neurite outgrowth and neuronal migration (Hori *et al.* 2014). Thus, it seems that AUTS2 is a multifunctional protein regulating distinct pathways in neural development. The roles of FBRS and FBRSL1 in neural development have not yet been explored.

We wanted to study the functions of *auts2* family genes in neural development using the zebrafish model system. Zebrafish provide multiple advantages for addressing these questions because of the ease of genetics, the transparency in early life stages, and the ability to image and record neural activity during nervous system development. As a first step toward this goal, in this manuscript, we describe the transcriptional complexity and the distinct expression patterns of *auts2* family genes in zebrafish.

## Materials and Methods

### Zebrafish use and care

Zebrafish (*Danio rerio*) of Indian wild-type strain were purchased from local suppliers and housed in aquarium tanks at 28° with a 14:10 hr light:dark cycle. Fish were maintained according to established protocols (Westerfield 2000) in agreement with the Institutional Animal Ethics Committee and the Institutional Biosafety Committee, National Centre for Biological Sciences.

### Sequence collection and gene structure annotation

*auts2* paralogs were identified using the Ensembl genome browser (GRCz10, Ensembl release 86). We manually curated sequences to extract intron–exon structural information. Gene models were retrieved from the Reference Sequence (RefSeq) database (NCBI *D. rerio* Annotation Release 105) and were validated experimentally by the sequence analysis of cDNA clones from our 5′-RACE (rapid amplification of cDNA ends) and RT-PCR experiments (see below). The putative alternative transcription start sites (TSSs) were identified using our 5′-RACE data and RNASeq gene models, generated from the Wellcome Trust Sanger Institute Zebrafish Transcriptome Sequencing Project, Ref: ERP000016 (Collins *et al.* 2012). The annotated RNASeq gene models are incorporated into the Ensembl genome browser (www.ensembl.org/Danio_rerio/). In our analysis, we assumed that if the position of the first nucleotide in RNASeq transcript is annotated in an intron, it can be considered as a putative TSS.

### Rapid amplification of 5′ cDNA ends (5′-RACE), RT-PCR, and cloning of the full-length cDNAs of auts2 paralogs

For 5′-RACE experiments, one microgram of a total RNA isolated from 24 hr embryos was utilized as a template for synthesis of first-strand cDNA using a SMARTer RACE cDNA amplification kit (Clontech) according to the manufacturer’s instructions. The 5′-RACE reactions were performed using the Advantage 2 Polymerase Mix (Clontech). Final PCR products were cloned into pCRII-TOPO vector (Invitrogen) and sequenced.

For RT-PCR analysis, total RNA was isolated from zebrafish embryos at different developmental stages using an RNeasy Mini kit (QIAGEN) and first-strand cDNAs were synthesized from 1 μg of a total RNA by oligo(dT) priming using SMARTScribe Reverse Transcriptase (Clontech) according to the manufacturer’s protocol. Amplification of cDNA was performed using Herculase II Fusion DNA polymerase (Agilent). Identity of amplified PCR products was verified by direct sequencing. The same batch of cDNAs was used to profile expression of *auts2* paralogs during development.

Full-length cDNAs of *auts2* paralogs were amplified using Q5 Hot Start High-Fidelity DNA Polymerase (New England Biolabs) and the resulting PCR products were cloned into pCR-Blunt vector (Invitrogen). Positive clones were verified by sequencing. Sequences of all primers used in this study will be provided upon request.

### RNA probe synthesis

The cDNA-containing vectors were linearized with appropriate restriction enzymes (detailed maps of vectors will be provided upon request) and used as a template for RNA probe synthesis. Sense and antisense RNA probes were synthesized using MEGAscript SP6 or T7 kits (Ambion) and either digoxigenin-labeled or fluorescein-labeled rNTPs (Roche).

### Whole-mount in situ hybridization (WISH)

Colorimetric WISH was conducted according to standard protocol (Thisse and Thisse 2008) with minor modifications. The protocol was identical for both embryos and whole brains. Briefly, embryos at different developmental stages were fixed in 4% paraformaldehyde in phosphate-buffered saline (PBS, pH 7.4) at 4° overnight. For expression analysis in the juvenile brain, 45-d-old fish were anesthetized in 0.02% Tricaine (ethyl 3-aminobenzoate methanesulfonate, Sigma) and then decapitated. The brains were dissected out from the skull, immediately immersed in cold 4% paraformaldehyde, and fixed at 4° for 24 hr. After fixation, embryos and brains were dehydrated in 100% methanol and stored at −20° until use. The methanol-stored embryos and brains were rehydrated in PBS containing 0.1% Tween-20 (PBST) and permeabilized by proteinase K treatment (Ambion, 10 μg/ml; incubation time was 10 min for brains and 18–24 hr embryos, and 20 min for 48 hr embryos; embryos younger than 18 hr were not treated with proteinase K). Hybridization was carried out in buffer [50% formamide, 5× SSC, 50 μg/ml heparin, 500 μg/ml tRNA (Roche), and 0.1% Tween-20] containing 5% dextran sulfate at 69° overnight. After stringency wash, the specimens were blocked with 2% blocking reagent (Cat. No. 11096176001; Roche) in maleic acid buffer (100 mM maleic acid, 150 mM NaCl, and 0.1% Tween-20, pH 7.5) and incubated overnight with anti-digoxigenin antibody, conjugated with alkaline phosphatase (1:5000; Roche) at 4°. For the colorimetric detection of alkaline phosphatase, specimens were incubated in staining buffer (100 mM Tris-HCl, 100 mM NaCl, 50 mM MgCl_2_, and 0.1% Tween-20, pH 9.5) containing 375 μg/ml nitro-blue tetrazolium chloride (Roche) and 175 μg/ml 5-bromo-4-chloro-3′-indolyl-phosphate (Roche).

**Figure 1:**
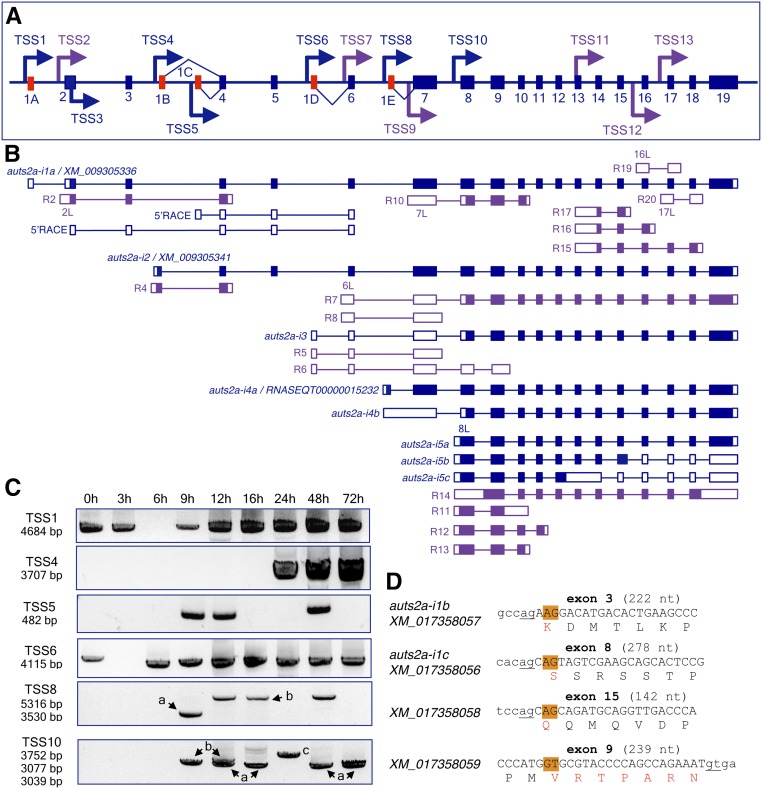
Gene structure and transcript complexity in the zebrafish *auts2a* gene locus. (A) Schematic presentation of the 560 kb long *auts2a* gene locus on chromosome 10 (not to scale). Exons are shown as bars. Mutually exclusive first exons (1A, 1B, 1C, 1D, and 1E) are in red. Arrows mark TSSs supported by either 5′-RACE and RT-PCR (blue) or RNASeq data (purple). TSSs are associated with PAPs. TSS3 was mapped inside of the coding region of exon 2. (B) Overview of transcripts generated in the *auts2a* gene locus. RNAs identified in this study and RNASeq transcripts from a public database are shown in blue and purple, respectively. Introns are shown as a line. Noncoding and coding exons are depicted as open and filled bars, respectively. RNASeq transcript’s IDs are shown in Table S2. Exons 2L, 6L, 7L, 8L, 16L, and 17L are 5′ extensions of the corresponding constitutive exons. (C) RT-PCR analysis of *auts2a* isoform expression during zebrafish development. Primers amplifying the full-length transcripts were used for analysis. Letters *a*, *b*, and *c* stand for the respective isoforms. (D) Partial DNA sequence of coding exons undergoing alternative splicing. Positions of alternative 5′ donor (exon 9) or 3′ acceptor (exons 3, 8, and 15) splice sites are highlighted in orange. Constitutive splice sites are underlined. Splicing at these sites leads to in-frame deletion of either single or seven amino acids (red) in the Auts2a protein. Exonic sequence is shown in upper case. Alternatively spliced isoforms identified in this study correspond to RefSeq transcripts (GenBank accession numbers are shown). ID, identifier; PAP, putative alternative promoter; RACE, rapid amplification of cDNA ends; RNASeq, RNA sequencing; RT-PCR, reverse transcription-polymerase chain reaction; TSS, transcription start site.

The following antisense and sense digoxigenin-labeled riboprobes were generated: (i) *auts2a*, 4.2 kb long probe corresponds to *auts2a-i3* isoform (structure of isoform can be found in Figure 1B), probe comprises 1508 bp of 5′-UTR, 2433 bp of open reading frame (ORF), and 174 bp of 3′-UTR; (ii) *fbrsl1*, 5.2 kb long probe corresponds to *fbrsl1-i2b* isoform (structure of isoform can be found in Figure 6B), probe comprises 1252 bp of 5′-UTR, 2529 bp of ORF, and 1291 bp of 3′-UTR; (iii) *fbrs*, 5.2 kb long probe comprises 780 bp of 5′-UTR, 3648 bp of ORF, and 677 bp of 3′-UTR; and (iv) *auts2b*, 2.5 kb long probe comprises 144 bp of 5′-UTR, 2247 bp of ORF, and 25 bp of 3′-UTR. Sense probes showed no specific or unspecific staining (see Supplemental Material, Figure S1).

cDNAs encoding *egr2a* (*krox20*) and *otx2* were kindly donated by Vladimir Korzh (International Institute of Molecular and Cell Biology, Warsaw); cDNAs encoding *wnt1* and *sim1a* were amplified and cloned into pGEM-TEasy (Promega) and pCRII-TOPO (Invitrogen) vectors, respectively. These cDNAs were used to generate fluorescein-labeled antisense riboprobes.

### Double in situ hybridization

To detect two riboprobes simultaneously, we used a two-color *in situ* hybridization protocol combining colorimetric and tyramide signal amplification-based fluorescent detection systems. The procedure for double *in situ* hybridization on embryos was essentially the same as that for colorimetric WISH, with the following modifications. (1) Prior to proteinase K treatment, embryos were incubated in 3% H_2_O_2_ in PBST for 30 min to quench endogenous peroxidase activity. (2) Digoxigenin-labeled and fluorescein-labeled riboprobes were mixed and used simultaneously during hybridization. (3) We first detected the fluorescein-labeled riboprobe using anti-fluorescein antibody, conjugated with horse-radish peroxidase (1:500; Roche). After washes, embryos were incubated for 30 min in the dark with the tyramide-Alexa Fluor 488 (Cat. No. T20948; Molecular Probes) working solution, prepared according to the manufacturer’s instructions. (4) The detection of the digoxigenin-labeled riboprobe was performed as for the colorimetric WISH using anti-digoxigenin antibody, conjugated with alkaline phosphatase. In case of fluorescent detection of the digoxigenin-labeled riboprobe, we first quenched peroxidase conjugated with anti-fluorescein antibody followed by incubation of embryos with anti-digoxigenin antibody conjugated with peroxidase (1:1000; Roche). After washes, embryos were incubated with the tyramide-Alexa Fluor 594 (Cat. No. T20950; Molecular Probes) working solution.

### Microscopy and imaging

Embryos were mounted in 70% glycerol for microscopy and imaging. The brains were embedded in 1.5% agar, blocks were saturated with 30% sucrose in PBS at 4° overnight, and then cut at 12 μm using a cryostat (Leica CM1850 UV). Light and fluorescent images were acquired using a stereomicroscope (OLYMPUS SZX16) equipped with a mercury lamp and digital camera (Jenoptik ProgRes C3). The images were processed using ImageJ (NIH) and Adobe Photoshop CS3.

### Data availability

All constructs, primers, and raw data are available upon request. Nucleotide sequences are deposited to GenBank under accession numbers KY492367–KY492385.

## Results

### Structure of the zebrafish auts2a gene locus

We initially performed bioinformatic analysis to evaluate the structure of the zebrafish *auts2a* gene locus. The current RefSeq *auts2a* gene model (NCBI Gene ID: 368890) defines 19 exons annotated in this genomic locus (exons 1A–19 in Figure 1A) and incorporates six computationally annotated transcripts. The RefSeq transcript XM_009305336 begins with untranslated exon 1A (Figure 1B) and codes for the long protein isoform with 1278 amino acid residues (see Figure S2A). Transcript XM_009305341 begins with the mutually exclusive exon 1B located in intron 3 and spliced to exon 4 (Figure 1B). This transcript codes for a protein of 1128 amino acid residues with 16 unique residues at the N-terminal end and lacking 166 N-terminal amino acid residues present in the long isoform (see Figure S2B). The other four RefSeq transcripts represent alternatively spliced variants of the long isoform XM_009305336. Alternative splicing occurs at tandem splice acceptors with a NAGNAG motif found at 3′ acceptor splice sites of exon 3 (transcript XM_017358057), exon 8 (transcript XM_017358056), and exon 15 (transcript XM_017358058). Alternative tandem splicing of mRNA leads to the exclusion of 3 nt (nucleotides) and, as a result, deletion of a single amino acid in the protein (Figure 1D). Splicing at an alternative 5′ donor splice site of exon 9 (transcript XM_017358059) leads to the exclusion of 21 nt from the spliced mRNA and, as a result, in-frame deletion of seven amino acids in the auts2a protein (Figure 1D).

In addition to the RefSeq transcripts, numerous RNASeq transcripts generated from the Wellcome Trust Sanger Institute Zebrafish Transcriptome Sequencing Project (Collins *et al.* 2012) are currently incorporated into the *auts2a* gene locus (for details visit www.ensembl.org/Danio_rerio/). The majority of RNASeq transcripts are apparently transcribed from unique TSSs and detected at specific developmental time points (summarized in Table S2). Analysis of RNASeq data revealed two additional mutually exclusive first exons, 1D and 1E, located in introns 5 and 6 and spliced to exons 6 and 7, respectively (Figure 1A). Transcript RNASEQT00000015232, identified from an olfactory epithelium RNA library (see Table S2), begins with exon 1E and encodes a protein of 1083 amino acid residues with 32 unique residues at the N-terminus. It lacks 227 residues in the N-terminus that are present in the long protein isoform (see Figure S2B). In the zebrafish genome assembly (GRCz10, Ensembl release 86), transcript ENSDART00000078920 corresponds to RNASEQT00000015232.

In addition, several alternative first exons that represent 5′ extensions of the corresponding constitutive exons, exon 2L (5′ extension of exon 2), exon 6L, exon 7L, exon 8L, exon 16L, and exon 17L, were identified from RNASeq data (Figure 1B and Table S2). TSSs associated with these exons are located in the flanking introns: intron 1 (TSS2), intron 5 (TSS7), intron 6 (TSS9), intron 7 (TSS10), intron 15 (TSS12), and intron 17 (TSS13) (Figure 1A, see Figure S3 for details). The exon beginning with TSS11 encompasses sequences of exon 13, intron 13, and exon 14 (Figure 1, see Figure S3J for details). The majority of mRNAs transcribed from these TSSs have alternative 3′-ends in introns and either encode small proteins or represent putative noncoding RNAs (see Table S2).

From *in silico* data analysis, we could define the presence of multiple putative TSSs that are apparently used to generate alternative *auts2a* mRNAs. Assuming that different TSSs are supposed to be associated with the corresponding putative alternative promoters (PAPs), we could predict, in addition to the major 5′ promoter associated with TSS1, the existence of multiple PAPs in the *auts2a* gene locus (Figure 1A).

### Identification of novel transcripts in the zebrafish auts2a gene locus

To clarify *in silico* data and to identify novel mRNAs transcribed from the *auts2a* gene locus, we performed 5′-RACE and RT-PCR analyses followed by isolation of full-length cDNAs encoding zebrafish *auts2a* mRNAs.

For 5′-RACE, we designed gene-specific primers in the 5′-part of the gene (in exons 4 and 6). Total RNA isolated from 24 hr embryos was used as a template in 5′-RACE experiments. We amplified 11 5′-RACE products. The majority of the 5′-ends were mapped in close proximity to the annotated positions of the first nucleotides of RNASeq or RefSeq transcripts (see Figure S3 for details). Sequence analysis of 5′-RACE products confirmed previously annotated alternative first exons 1A, 1B, and 1D, and identified a novel mutually exclusive first exon 1C located in intron 4, placed 51,016 nt downstream from exon 1B (Figure 1A). Exon 1C is spliced to exon 4, similar to exon 1B, and no overlapping RefSeq or RNASeq annotations were found for this exon. When we used a reverse primer designed in the 3′-UTR of exon 19, we could not amplify cDNA beginning with exon 1C. However, the transcript corresponding to the 5′-RACE product could be detected by RT-PCR during analysis of *auts2a* expression through zebrafish development (TSS5 in Figure 1C), suggesting that RNA transcribed from TSS5 has an alternative 3′-end.

We noticed a few interesting features when we analyzed the 5′-RACE results. First, multiple 5′-ends of different lengths were mapped into exons 1A (five 5′-ends) and 1D (four 5′-ends) (see Figure S3, A and E). The mapping was in close proximity to the annotated positions of the first nucleotides of RNASeq and/or RefSeq transcripts. Remarkably, all TSSs in exon 1A (annotated and experimentally determined) were clustered within 58 nt, while TSSs in exon 1D were spread over 300 nt. From genome-wide analysis of mammalian promoters, it was found that sharp starting sites are generally associated with promoters having TATA-boxes, while promoters associated with CpG islands do not show an accurate TSS, but instead a broad distribution of TSSs generally spread over 100 nt (Carninci *et al.* 2006; Gustincich *et al.* 2006). We found a TATA-box motif in the promoter associated with TSS1 (see Figure S3A), but not in the promoter associated with TSS6. Except for the brain, where TSSs are surprisingly enriched in CpG islands, TATA-box-promoted transcripts tend to be tissue-specific (Gustincich *et al.* 2006). Despite this fact, the presence of multiple TSSs mapped to a single exon could also be accounted for by random transcription initiation events from the same promoter; even a small extension in the 5′-end sequence may also play an important role, for example, by harboring upstream ATGs (and upstream ORFs) that throttle the translation from the downstream authentic ATG.

Second, in one case we mapped a 5′-end into the coding region of exon 2 (TSS3 in Figure 1A), 603-bp downstream from the first nucleotide of transcript RNASEQT00000008207 annotated in intron 1 (this transcript begins with alternative exon 2L, see Figure S3B). It is generally assumed that the range over which the TSSs are scattered is on average 62 bp (Suzuki *et al.* 2001) and that two independent TSS clusters, associated with distinct PAPs, are separated with > 500 bp intervals (Kimura *et al.* 2006). Since TSS3 was mapped at a distance > 500 bp from the first nucleotide of the RNASeq transcript, it is likely that TSS3 is associated with a different PAP, perhaps overlapping with that of TSS2. Although the location of TSS within the internal exon could be accounted for by the truncated cDNAs, it could also be a genuine “internal” putative promoter with unique features different from canonical promoters since the corresponding genomic regions serve as both exon and promoter. Interestingly, genes having TATA-box promoters are also preferentially associated with the presence of unusual transcripts, originating from exons (Carninci *et al.* 2006).

To amplify full-length cDNAs that begin with alternative first exons, we designed a set of exon-specific forward primers that bind in close proximity to the annotated TSSs (see Figure S3 for positions of individual primers). Since reverse primers were bound to the 3′-UTR of exon 19, we could only amplify the population of cDNAs with complete ORFs that differ in the 5′-end but were similar in the 3′-end. With such an approach, the presence of alternative 3′-ends was not explored. The same set of primers was used for RT-PCR analysis of *auts2a* expression during zebrafish development.

We cloned four variants of the long isoform *auts2a-i1*, all beginning with exon 1A and corresponding to the RefSeq transcripts: (1) *auts2a-i1a* (XM_009305336), (2) *auts2a-i1b* (XM_017358057), and (3) *auts2a-i1c* (XM_017358056). In the fourth transcript variant *auts2a-i1d*, noncoding exon 1A was spliced to exon 2 by utilizing a cryptic 5′ donor splice site located in the intron, 132 nt downstream from the constitutive splice site (see Figure S3A). This leads to the formation of a transcript with different 5′-UTR length but does not affect the coding region of *auts2a*. Transcript *auts2a-i1d* also has an alternatively spliced exon 8, similar to isoform *auts2a-i1c*. We did not clone transcript variants corresponding to XM_017358059 (alternative exon 15) and XM_017358058 (alternative exon 9). However, direct sequencing of PCR products revealed coamplification of transcripts with constitutively and alternatively spliced exons 3, 8, and 15. RT-PCR analysis showed that the *auts2a-i1* isoform is maternally supplied and is also present from 9 to 72 hr (the last examined time point; TSS1 in Figure 1C).

Transcript isoform *auts2a-i2* beginning with exon 1B corresponds to XM_009305341 (Figure 1B), with the exception that, in contrast to the RefSeq transcript, cDNA of *auts2a-i2* has both alternatively spliced exons 8 and 15. During zebrafish development, *auts2a-i2* was detected only at 24, 48, and 72 hr (TSS4 in Figure 1C).

Transcript isoform *auts2a-i3* begins with untranslated exon 1D and contains alternatively spliced exons 8 and 15 (Figure 1B). Direct sequencing of PCR products revealed coamplification of transcripts with constitutively and alternatively spliced exons 8 and 15, similar to the long isoform *auts2a-i1*. Exon 8 in isoform *auts2a-i3* consists of 181 nt of the 5′-UTR and 94 nt of CDS, and splicing at an alternative 3′ acceptor splice site in this isoform does not affect protein sequence as it does in isoform *auts2a-i2* (deletion of serine). The ORF of this transcript is identical to that of the long isoform except that it lacks 467 amino acid residues in the N-terminus (Figure S2A). Similar to isoform *auts2a-i1*, *auts2a-i3* is maternally supplied and then is detected from 6 to 72 hr during zebrafish development (TSS6 in Figure 1C).

Two novel transcript isoforms, *auts2a-i4a* and *auts2a-i4b* beginning with exon 1E, were isolated during RT-PCR analysis. In contrast to the annotated transcript RNASEQT00000015232, exon 1E in isoform *auts2a-i4a* has 85 nt of 3′ extension due to the usage of alternative 5′ donor splice site (see Figure S3G). Isoform *auts2a-i4a* encodes a protein of 1082 amino acid residues with a unique stretch of nine residues at the N-terminal end *vs.* 32 unique residues in protein coded by RNASEQT00000015232 (see Figure S2B). In isoform *auts2a-i4b*, the first exon retains 1786 nt of intronic sequence and extends through exon 7. Despite this intron retention, the ORF of *auts2a-i4b* is identical to that of the *auts2a-i3* isoform. However, the sequence of the 5′-UTR (2880 nt long) in this isoform differs from that in isoform *auts2a-i3* (5′-UTR of 1684 nt long). During zebrafish development, isoform *auts2a-i4a* was detected exclusively at 9 hr, while *auts2a-i4b* could be detected at 12, 16, and 48 hr (TSS8 in Figure 1C). We could not isolate cDNA with the structure of exon 1E corresponding to RNASEQT00000015232. Since this RNASeq transcript was found in sample prepared from olfactory epithelium of adult fish (see Table S2), it may explain why we could not amplify it during our RT-PCR analysis.

RNASeq data show two transcripts beginning with exon 8L (5′ extension of exon 8) having alternative 3′-ends and encoding small proteins of 219 and 196 amino acid residues with unique N- and C-terminal ends (see Figure 1B and Table S2). During RT-PCR analysis, we could isolate three novel isoforms beginning with exon 8L that share their 3′-ends with the long isoform *auts2a-i1*. Isoform *auts2a-i5a* encodes a protein of 888 amino acid residues with 15 unique residues at the N-terminal end (Figure 1B and Figure S2B). Two other isoforms represent alternatively spliced transcripts with retention of intronic sequences that lead to premature stop codons. Isoform *auts2a-i5b* retains 38 nt from intron 15, while isoform *auts2a-i5c* retains 745 nt from introns 12 and 13, including exon 13. During zebrafish development, isoform *auts2a-i5a* was detected at 12, 16, 48, and 72 hr; isoform *auts2a-i5b* at 9 and 12 hr; and isoform *auts2a-i5c* at 24 hr only (TSS10 in Figure 1C).

### Expression of auts2a mRNA during zebrafish development

We conducted WISH to examine how the spatial expression patterns of *auts2a* transcripts vary during development. Two isoforms, *auts2a-i1* and *auts2a-i3*, were expressed through all developmental stages according to RT-PCR analysis (Figure 1C). Since the usage of an antisense RNA probe detecting full-length *auts2a-i1* isoform showed ubiquitous staining (data not shown), we used an antisense probe detecting full-length *auts2a-i3* isoform for analysis, which showed more restricted staining. For probe details see *Materials and Methods*.

**Figure 2:**
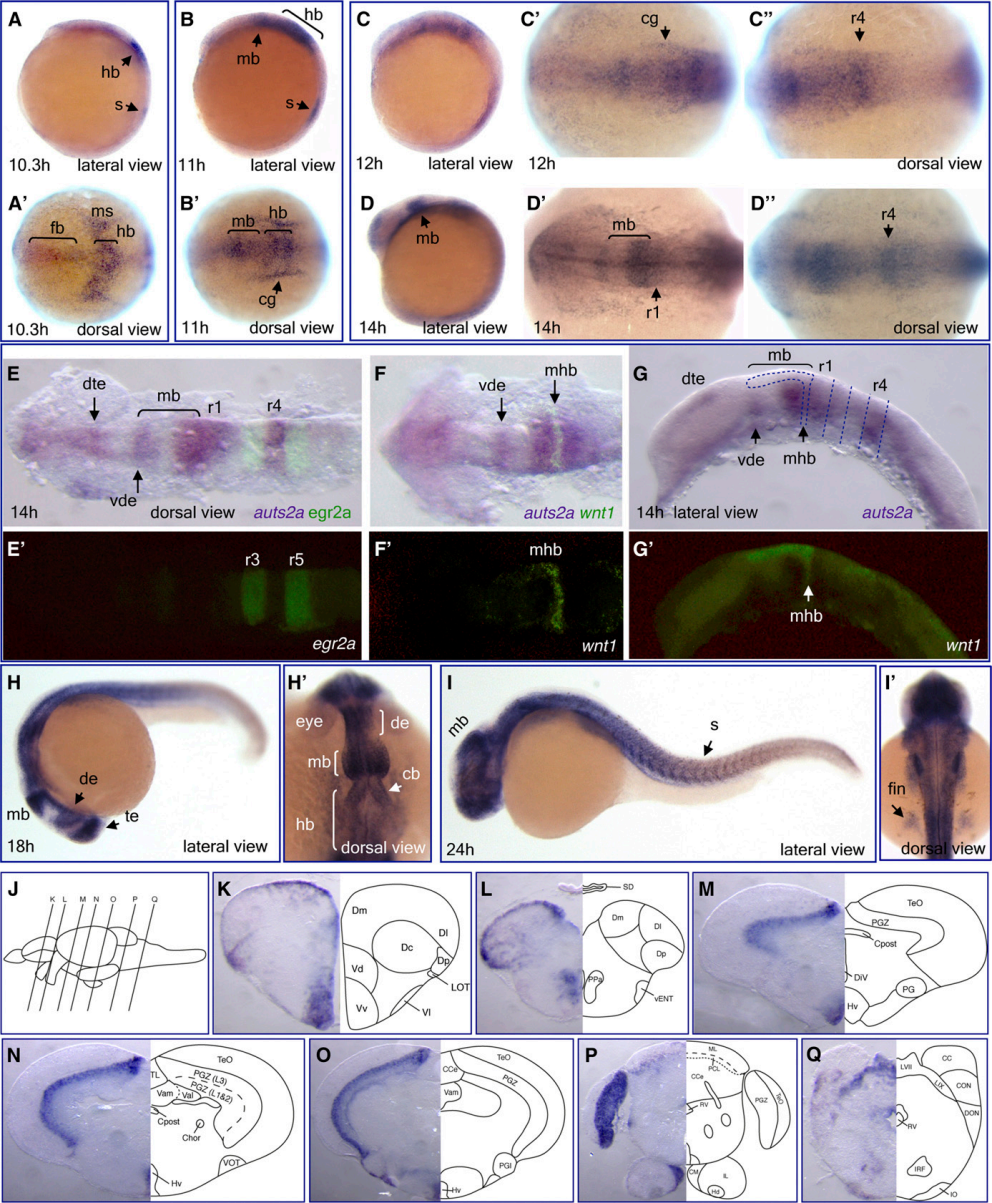
Expression of *auts2a* mRNA during zebrafish development. Whole-mount *in situ* hybridization analysis of *auts2a-i3* transcript expression in wild-type embryos at different developmental stages: 10.3 hr (A and A’), 11 hr (B and B’), 12 hr (C–C”), 14 hr (D–D”), 18 hr (H and H’), and 24 hr (I and I’). (E–G’) Double *in situ* hybridization with *egr2a* (*krox20*) (E and E’) and *wnt1* (F’ and G’) as second probes. In (G), dotted lines approximately demarcate rhombomere borders and midbrain. (K–Q) Transverse sections at the levels indicated by the vertical lines in (J) show *auts2a-i3* mRNA expression in different regions of the juvenile brain: telencephalon (K and L), diencephalon and midbrain (M–O), and hindbrain (P and Q). Abbreviations used to label areas in brain sections can be found in Table S1. cb, cerebellum; cg, cranial ganglia; de, diencephalon; dte, dorsal telencephalon; fb, forebrain; hb, hindbrain; mb, midbrain; mhb, midbrain-hindbrain boundary; ms, mesoderm; r, rhombomere; s, somite; te, telencephalon; vde, ventral diencephalon.

Weak expression of *auts2a-i3* is first detected at the 1-somite stage (10.3 hr) in a medial part of the presumptive forebrain and more intensely in the presumptive hindbrain. In the hindbrain region, *auts2a-i3* expression extends into the mesoderm laterally to the neural plate. It is also expressed in the somite (Figure 2, A and A’). At the 3-somite stage (11 hr), *auts2a-i3* continues to be weakly expressed in the presumptive forebrain and more intensely in the presumptive midbrain and hindbrain. It is also expressed outside of the neural plate, in two longitudinal stripes lateral to the hindbrain, presumably the primordial cranial ganglia, and in somites (Figure 2, B and B’). During neural keel formation (12–14 hr), weak expression of *auts2a-i3* is continued in the forebrain and more intensely in midbrain and hindbrain, with stronger expression in presumed rhombomere 4 (Figure 2, C–C” and D–D”). To demarcate *auts2a* expression in the brain at this developmental stage, we performed double *in situ* hybridization using an *egr2a* (*krox20*) probe to mark rhombomeres 3 and 5 (Figure 2E’), and a *wnt1* probe to mark the midbrain–hindbrain boundary (Figure 2, F’ and G’). Indeed, *auts2a-i3* was expressed in the telencephalon with more intense expression in the dorsal part, and ventrally in the rostral diencephalon (Figure 2G), posterior midbrain, rhombomeres 1 and 4, and the spinal cord (Figure 2, E, F, and G). At 18 hr, *auts2a-i3* is expressed in the telencephalon, diencephalon, midbrain, hindbrain, and spinal cord. It is also expressed in somites (Figure 2, H and H’). At 24 hr, *auts2a-i3* is expressed in the brain with stronger expression in the tectum. It is also expressed in the spinal cord, somites, and pectoral fin buds (Figure 2, I and I’).

We also performed *in situ* hybridization analysis of *auts2a-i3* expression in the juvenile zebrafish brain (Figure 2, J–Q). In the forebrain, *auts2a-i3* expression is detected in the ventral telencephalic area with strong expression in the ventral nucleus (Vv) and anterior part of the parvocellular preoptic nucleus (PPa), as well as in the dorsal telencephalic area with strong expression in posterior (Dp) and medial parts (Dm) (Figure 2, K and L). Expression of *auts2a-i3* is also detected in the mammillary body (CM) (Figure 2P), a part of the hypothalamus that is important for recollective memory in rodents (Vann 2010), and in the periventricular zone of the hypothalamus (Hv) (Figure 2M). In the midbrain, *auts2a-i3* expression is primarily detected in layer three of the periventricular gray zone of the optic tectum (PGZ) (Figure 2, M–O). In the cerebellum, *auts2a-i3* is expressed in the Purkinje cell layer (PCL) (Figure 2P); in the hindbrain, expression is detected dorsally in facial (LVII) and glossopharyngeal (LIX) lobes, the caudal octavolateralis nucleus (CON), around the rhombencephalic ventricle (RV); and ventrally, in the inferior reticular formation (IRF) and inferior olive (IO) (Figure 2Q).

Since multiple transcript isoforms were identified in the *auts2a* gene locus, we wanted to determine if these isoforms are differentially expressed during development. To evaluate isoform-specific expression of *auts2a* transcripts, we designed riboprobes that recognize alternative first exons: mutually exclusive exons (see Figure S4A). For *in situ* hybridization, we chose 24 hr embryos as this was the earliest stage at which three of the *auts2a* isoforms were expressed (Figure 1C). We could detect reliable expression signals for *auts2a* transcripts generated from TSS1 and TSS6, but not for that from TSS4 (Figure S4B), probably because of the short length of the probe (204 bp). The expression pattern of *auts2a* isoforms generated from TSS1 and TSS6 was quite similar in the brain, with stronger expression of the TSS6 isoform in the telencephalon and midbrain (tectum) in comparison to the TSS1 isoform (Figure S4B).

### Duplication of the auts2 gene in teleost genomes

**Figure 3:**
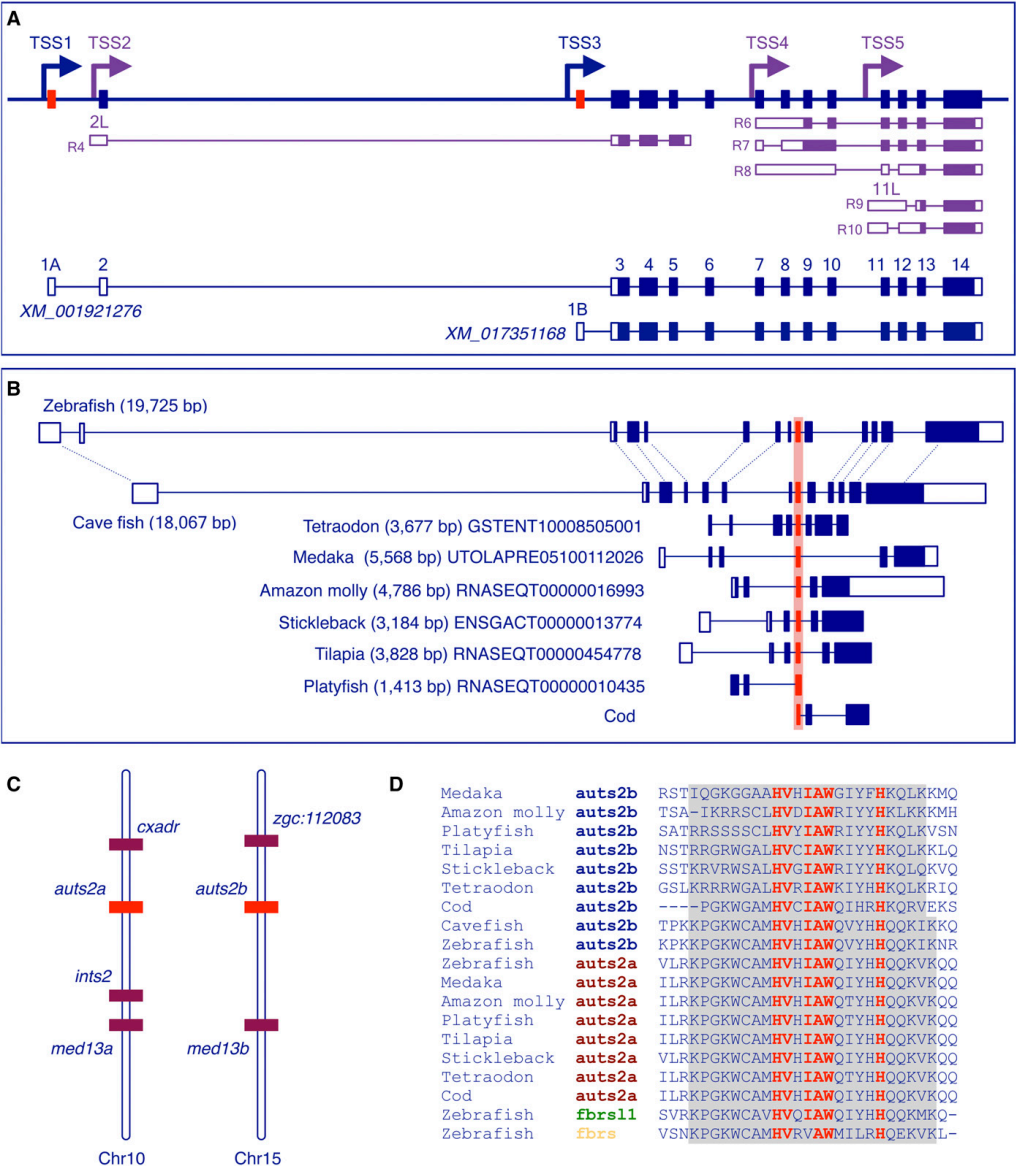
Gene structure and transcript complexity in the zebrafish *auts2b* gene locus. (A) Schematic presentation of the 20 kb long *auts2b* genomic region on Chr15 (not to scale). Exons are shown as bars. Mutually exclusive first exons (1A and 1B) are in red. Arrows show TSSs identified either in this study (blue) or annotated based on RNASeq data (purple). Structures of RNASeq transcripts transcribed from alternative TSSs are shown. RNASeq transcript IDs are shown in Table S3. Exons 2L and 11L are 5′ extensions of the corresponding exons. Noncoding and coding exons are depicted as open and filled bars, respectively. (B) Schematic presentation of the structure of *auts2b* genes in genomes of different teleosts. Conserved exon 9 is depicted in red. (C) Schematic showing synteny between *auts2a* and *auts2b* genomic regions in the zebrafish genome. (D) Alignment of amino acid sequence coded by conserved exons 9 (Auts2b) and 14 (Auts2a) from different teleosts (coding exon is highlighted in gray). Amino acids that are highly conserved among Auts2 paralogs are shown in red. Chr, chromosome; ID, identifier; RNA sequencing; TSS, transcription start site.

During evolution, teleost genomes experienced an additional round of whole-genome duplication (Christoffels *et al.* 2004; Hoegg *et al.* 2004). As a result, the zebrafish genome contains two *auts2* genes, *auts2a* on chromosome 10 and *auts2b* on chromosome 15 (Figure 3C). The current RefSeq *auts2b* gene model (NCBI Gene ID: 100150849) defines 14 exons present in transcript XM_001921276 (Figure 3A). In addition, transcript XM_017351168 represents an alternatively spliced variant with a mutually exclusive first exon 1B, which is located in intron 2 and spliced to exon 3 (Figure 3A). Analysis of RNASeq data allowed the prediction of an additional three alternative first exons representing 5′ extensions of constitutive exons: exon 2L (TSS2), exon 6L (TSS4), and exon 11L (TSS5) (summarized in Figure 3A and Table S3).

We performed 5′-RACE with gene-specific primers designed in exons 3 and 4. We isolated a single 5′-RACE product and mapped it in exon 1, 588 nt downstream from the first nucleotides of RNASeq transcript RNASEQT00000017723 (see Figure S5), which is not annotated in the current Ensembl release, but was included in the previous genome build (Zv9, Ensembl release 75). With a forward primer, located 217 nt downstream from the first nucleotide of RNASEQT00000017723, we isolated full-length cDNA structurally corresponding to XM_001921276 and ENSDART00000161379. During the sequence analysis of clones, we also identified a novel transcript with intron retention (106 bp of intron 5) leading to a premature stop codon.

Based on structure comparison between *auts2a* and *auts2b* genes, we assumed that, after whole-genome duplication, the 5′ genomic region of the ancestral gene corresponding to exons 1A–6 of *auts2a* was subsequently lost from the *auts2b* gene locus. Surprisingly, *in silico* analysis revealed the absence of the full-length *auts2b* gene in other teleosts whose genomes were sequenced (amazon molly, cod, fugu, medaka, platyfish, stickleback, tetraodon, and tilapia) with one exception. The genome of cave fish (*Astyanax mexicanus*) contains the *auts2b* gene but lacks the *auts2a* gene. It is not clear if *auts2a* gene is absent in the cave fish genome or it is simply not annotated in a current version of the cave fish whole-genome assembly due to incomplete sequencing. Protein sequence comparison and analysis of synteny strongly supports the identification of the cave fish *auts2* gene as an *auts2b* ortholog. We analyzed genomic regions in other teleost genomes that are syntenic to the *auts2b* locus in zebrafish genome. With the exception of the fugu genome, the other loci possess the predicted protein coding gene in the position of the *auts2b* gene. Protein sequence and gene structure analysis revealed a single conserved exon. This exon corresponds to exon 9 in the *auts2b* gene (Figure 3B). Exon 9 encodes 24 highly conserved amino acids within the Auts2 family domain (a single amino acid substitution in the Auts2a protein among teleosts and 100% identity in the Auts2b protein between zebrafish and cave fish) (Figure 3D). The rest of the amino acid sequence is highly diverse with low sequence similarity between analyzed proteins. At least in some teleosts this “gene” is transcribed as it is supported by RNASeq data from amazon molly, platyfish, and tilapia.

### Expression of the auts2b gene during zebrafish development

**Figure 4:**
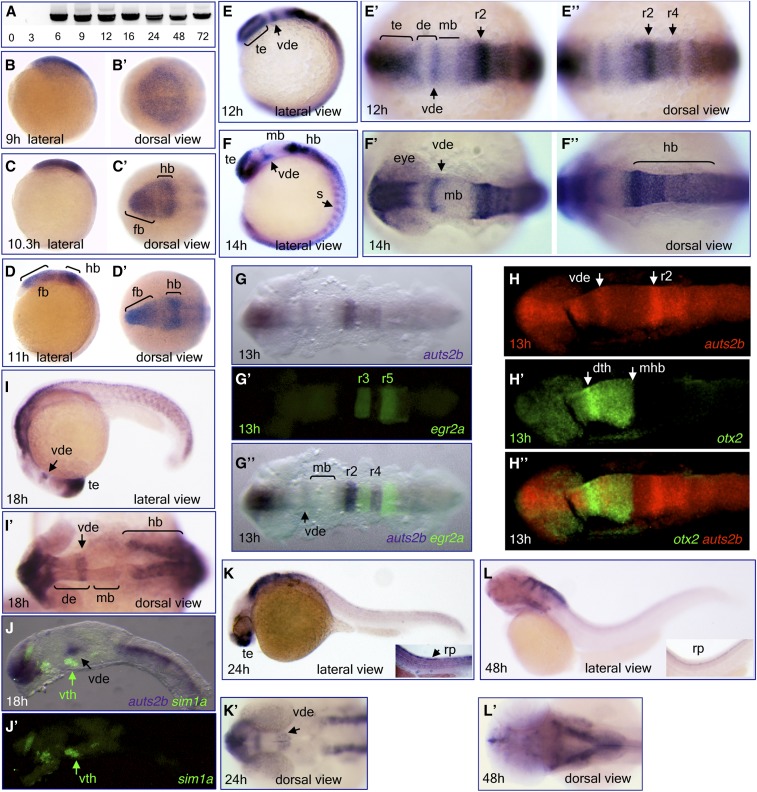
Expression of *auts2b* mRNA during zebrafish development. (A) RT-PCR analysis of *auts2b* expression during development (amplicon size is 3.25 kb). Numbers shown below each lane indicate sample age in hours post fertilization. (B–F”, I, I’, and K–L’): Whole-mount *in situ* hybridization analysis of *auts2b* transcript expression in wild-type embryos at different developmental stages: 9 hr (B and B’), 10.3 hr (C and C’), 11 hr (D and D’), 12 hr (E–E”), 14 hr (F–F”), 18 hr (I and I’), 24 hr (K and K’), and 48 hr (L and L’). (G–H”, J, and J’) Double *in situ* hybridization with *egr2a* (*krox20*) (G–G”), *otx2* (H–H”), and *sim1a* (J and J’) as second probes. de, diencephalon; dth, dorsal thalamus; fb, forebrain; hb, hindbrain; mb, midbrain; mhb, midbrain-hindbrain boundary; r, rhombomere; rp, roof plate; RT-PCR, reverse transcription-polymerase chain reaction; s, somites; te, telencephalon; vde, ventral diencephalon; vth, ventral thalamus.

RT-PCR analysis shows that *auts2b* is a zygotic gene in contrast to maternally supplied *auts2a* (Figure 4A). At 90% of epiboly (9 hr), *auts2b* is expressed in the rostral neural plate, the prospective forebrain territory (Figure 4, B and B’). At the 1-somite stage (10.3 hr), *auts2b* is expressed in the presumptive forebrain, presumptive hindbrain, and spinal cord (Figure 4, C and C’). At the 3-somite stage (11 hr), *auts2b* is strongly expressed in the forebrain, hindbrain, and faintly in the midbrain (Figure 4, D and D’). During neural keel formation (12–14 hr), *auts2b* is expressed in the telencephalon, ventral diencephalon, hindbrain (more strongly in rhombomere 2 and less in rhombomeres 1 and 4), spinal cord, and somites (Figure 4, E–F”). The identity of rhombomeres was confirmed by using second probes, *egr2a* (*krox20*) to mark rhombomeres 3 and 5 (Figure 4, G–G”), and *otx2* to mark the midbrain territory (Figure 4, H–H”). Expression of *auts2b* was not detected in the midbrain (Figure 4, F, F’, and G). At 18 hr, expression of *auts2b* continued in the telencephalon, ventral diencephalon, hindbrain, spinal cord, and somites (Figure 4, I and I’). Expression in the ventral diencephalon at this stage was suggested by double *in situ* hybridization with *sim1a* as a marker for the ventral thalamus (Figure 4, J and J’). At 24 hr, *auts2b* is expressed in the telencephalon, ventral diencephalon, and hindbrain (Figure 4, K and K’), and in the roof plate of the spinal cord (Figure 4K inset). At 48 hr, *auts2b* is mainly expressed in the dorsal hindbrain and cerebellum (Figure 4, L and L’), and in the roof plate (Figure 4L inset).

**Figure 5:**
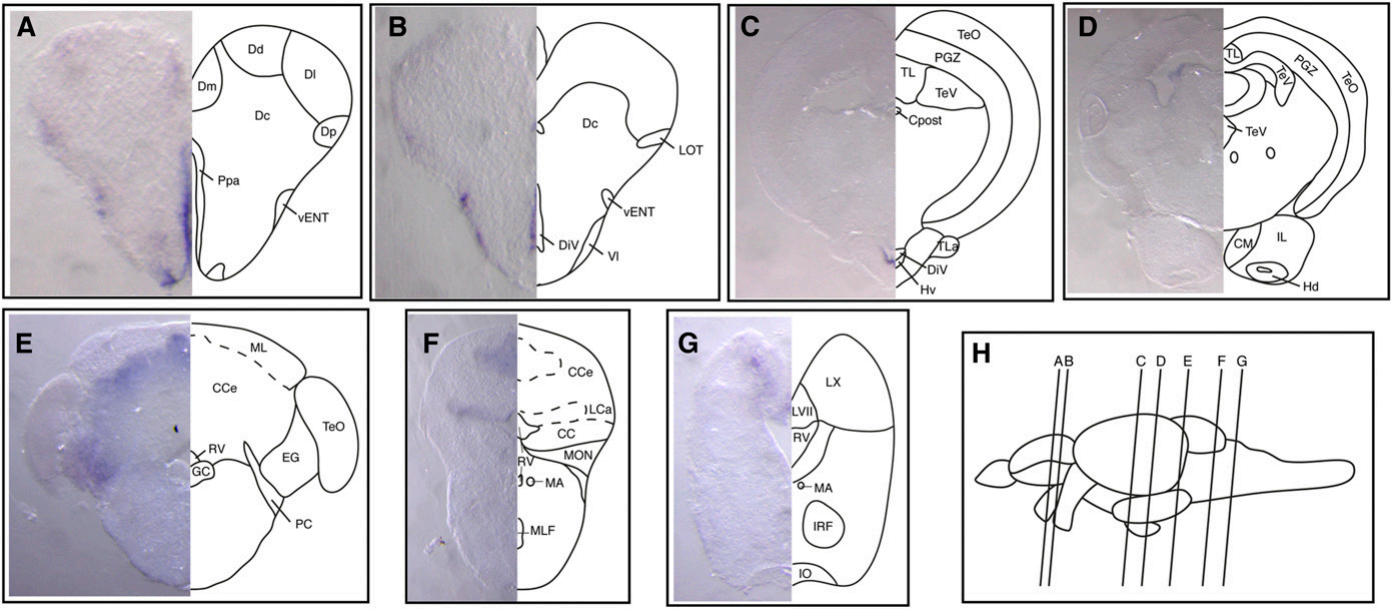
Expression of *auts2b* mRNA in juvenile brain. (A–G) Transverse sections at the levels indicated by the vertical lines in (H) show *auts2b* mRNA expression in different regions of the juvenile brain: telencephalon (A and B), diencephalon and midbrain (C and D), and hindbrain (E–G). Abbreviations used to label areas in brain sections can be found in Table S1.

We also performed *in situ* hybridization analysis of *auts2b* expression in the juvenile zebrafish brain (Figure 5). In the forebrain, expression of *auts2b* was detected in Dp and Dm zones of the dorsal telencephalic area, the anterior part of the PPa, the ventral entopeduncular nucleus (vENT), the lateral nucleus of ventral telencephalic area (Vl) (Figure 5, A and B), and the ventral zone of the periventricular hypothalamus (Hv) (Figure 5C). In the midbrain, *auts2b* expression was not detected (Figure 5, C and D). In the cerebellum, *auts2b* expression was observed in the granule cell layer of the corpus cerebelli (CCe) and eminentia granularis (EG) (Figure 5E). More caudally, *auts2b* is expressed in the caudal lobe of the cerebellum (LCa), a granular layer of EG (Figure 5F). In the hindbrain, expression of *auts2b* is detected in LVII and vagal (LX) lobes (Figure 5G).

### Identification of the 5′-ends of the zebrafish fbrsl1 gene and isolation of novel fbrsl1 transcripts

**Figure 6:**
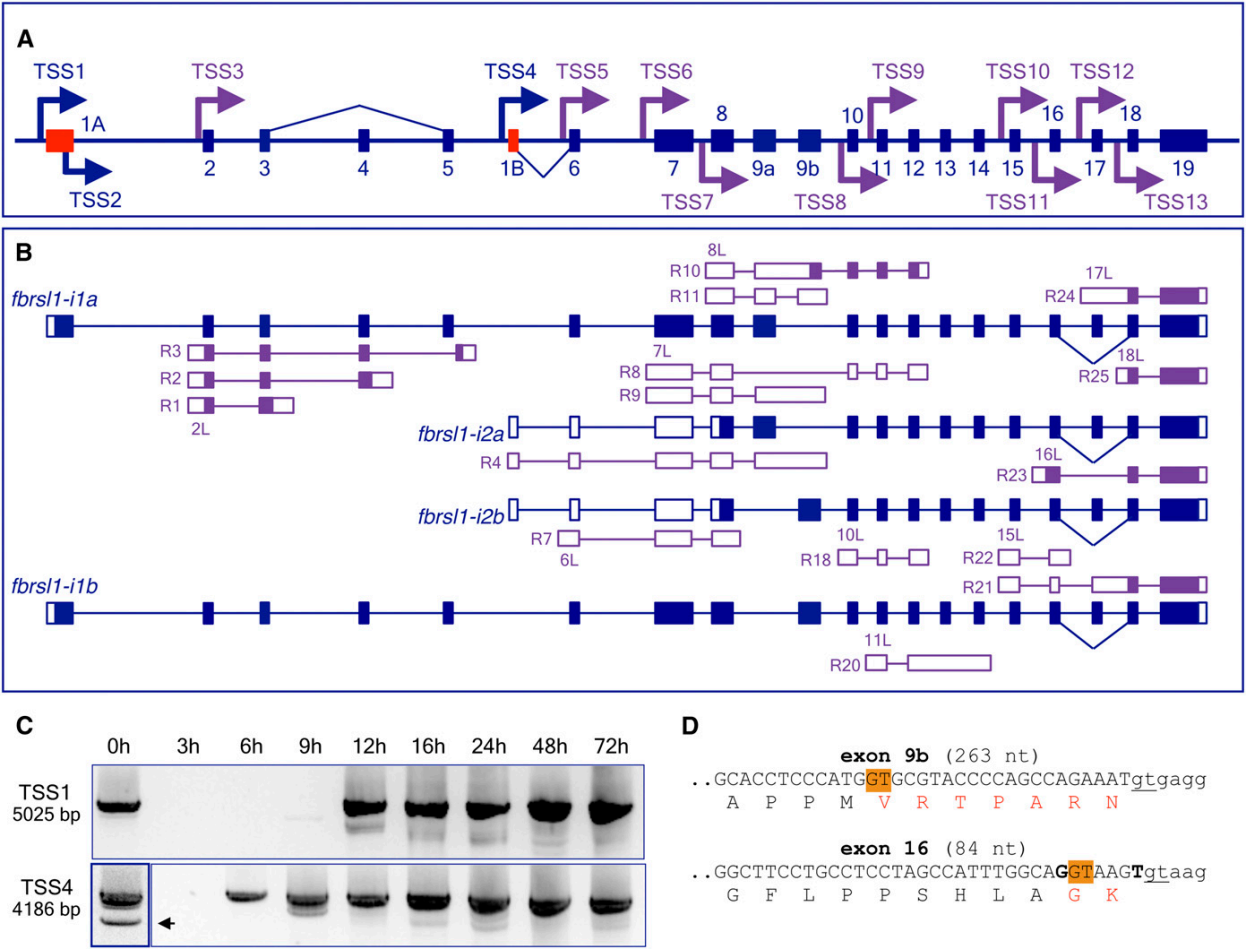
Gene structure and transcript complexity in the zebrafish *fbrsl1* gene locus. (A) Schematic presentation of the 580 kb long *fbrsl1* genomic region on chromosome 5 (not to scale). Exons are shown as bars. Mutually exclusive first exons (1A and 1B) are in red. Exons 9a and 9b are mutually exclusive internal exons. Arrows show TSSs identified either in this study (blue) or annotated based on RNASeq data (purple). TSS2 is found inside of the coding part of exon 1A. (B) Schematic presentation of the structure of *fbrsl1* transcripts identified in this study. Noncoding and coding exons are depicted as open and filled bars, respectively. Structure of RNASeq transcripts transcribed from alternative TSSs are shown. RNASeq transcript IDs are shown in Table S4. Exons 2L, 6L, 10L, 11L, 15L, and 17L are 5′ extensions of the corresponding exons. (C) RT-PCR analysis of *fbrsl1* isoforms expression during zebrafish development. Primers amplifying the full-length transcripts were used for analysis. The second band (pointed by arrow) is a reverse transcriptase template switching artifact. The 0 hr lane for TSS4 was taken from a different gel. (D) Partial DNA sequence of coding exons undergoing alternative splicing. Position of alternative 5′ donor splice sites is highlighted in orange. Constitutive splice sites are underlined. Exonic sequence is shown in upper case. Deleted amino acids are shown in red. ID, identifier; RNASeq, RNA sequencing; RT-PCR, reverse transcription-polymerase chain reaction; TSS, transcription start site.

In contrast to the *auts2a* gene, the RefSeq *fbrsl1* gene model (NCBI Gene ID: 557358) lacks a completely annotated 5′-end. Nevertheless, similar to the *auts2a* gene locus, several RNASeq transcripts coding for short polypeptides or representing noncoding RNAs are annotated in the *fbrsl1* gene locus (summarized in Figure 6A, for more details see Table S4 and the Ensembl browser). To define the 5′-end of the *fbrsl1* gene, we performed 5′-RACE analysis with gene-specific primers designed to exons 4, 5, and 7. We isolated 11 different 5′-RACE products and annotated two novel exons associated with them (see Figure S6, A–C).

Exon 1A contains 850 nt of 5′-UTR and 273 nt of CDS (see Figure S6A). Using BLAST, we could only map 1–548 nt to contig CU929333.5 (chromosome 5: 16,669,728–16,670,275 in Ensembl genomic coordinates), 135,534 nt upstream from the first nucleotide of RefSeq transcript XM_009301264. However, when we searched against the NCBI whole-genome shotgun database, this exon could be completely mapped to Zebrafish Genome Assembly WGS32 contig_000055998 (GenBank: CZQB01055999.1) at position 9394–10,516 (contig length is 54,890 bp). This contig is overlapped by contigs CABZ01050979.1 and CU929333.5 from Ensembl, although the gaps in sequence still remain (overlapping nucleotides 1–9395 and 9964–54,890).

From 5′-RACE experiments, six 5′-ends were mapped to the untranslated region of exon 1A and one 5′-end into the translated region of exon 1A, 214 nt downstream from ATG (see Figure S6A). Mapped TSSs were clustered in two groups (five 5′-ends in one group and two 5′-ends in the other) separated by 593 bp. Based on the idea that two independent TSS clusters associated with distinct PAPs are separated with > 500-bp intervals (Kimura *et al.* 2006), we predict that these two groups of TSSs are linked to distinct PAPs, likely overlapping, and can be recognized as separate TSSs: TSS1 and TSS2 (Figure 6B).

Using a forward primer designed in close proximity to the most distant 5′-end (see Figure S6A), we could isolate two transcripts, both beginning with exon 1A and including 19 exons. The difference between them is that the two isoforms, *fbrsl1-i1a* and *fbrsl1-i1b*, contain mutually exclusive exons 9a and 9b, respectively (Figure 6B). Transcripts *fbrsl1-i1a* and *fbrsl1-i1b* code protein isoforms of 1215 and 1249 amino acid residues, respectively (see Figure S6). The RefSeq transcript XM_009301264, although lacking exon 1A, apparently represents an alternative splice variant of *fbrsl1-i1b* with exon 9b being spliced at an alternative 5′ donor splice site leading to the exclusion of 21 nt from the spliced mRNA and, hence, an in-frame deletion of seven amino acids in the protein (Figure 6D). Transcript XM_009301264 also has an alternative exon 16, which is spliced at an alternative 5′ donor splice site leading to the exclusion of 6 nt from mRNA and therefore an in-frame deletion of two amino acids (glycine and lysine) and substitution of tyrosine with aspartic acid (Figure 6D and Figure S6). Notably, splicing at the alternative 5′ donor splice site of exon 9b is exactly the same as that in exon 9 of the XM_017358059 transcript from the *auts2a* gene locus (with deletion of identical amino acids).

The second novel exon, noncoding exon 1B identified in intron 5, is spliced to exon 6 (Figure 6A). We mapped three 5′-ends of different lengths to this exon (see Figure S6C). In a previous zebrafish genome build (Zv9, Ensembl release 75), RNASeq transcript RNASEQT00000006457 was annotated with exon 1B and its first nucleotide was 145 nt upstream from the TSS mapped in this study (see Figure S6C). In the current zebrafish genome assembly (GRC10) this transcript is not included. However, we could isolate cDNA *fbrsl1-i2b* corresponding to RNASEQT00000006457 (Figure 6B). Transcript *fbrsl1-i2b* includes mutually exclusive exon 9b and codes for a short protein isoform. The ORF of this short transcript is identical to that of the long isoform except that it lacks 405 N-terminal amino acids present in the long isoform, and is predicted to encode a polypeptide of 842 amino acid residues (see Figure S7). Ensembl transcript ENSDART00000081064 and RNASeq transcript RNASEQT00000054612, which lack the annotated exon 1B, apparently represent alternative splice isoforms of the *fbrsl1-i2a* transcript with mutually exclusive exon 9a and encode a protein of 810 amino acid residues (see Figure S7).

With a similar approach as was used for the *auts2a* gene locus, by using RNASeq data we defined additional 10 alternative first exons representing 5′ extensions of annotated exons: exon 2L (5′ extension of exon 2), exon 6L, exon 7L, exon 8L, exon 10L, exon 11L, exon 15L, exon 16L, exon 17L, and exon 18L (Figure 6, A and B and Figure S6). Several RNASeq transcripts have the first nucleotides annotated inside of mutually exclusive exons 9a and 9b, so the transcripts begin with ATG (see Table S4 for details). We did not consider such candidates as putative TSSs because it could be accounted for by truncated cDNAs. However, we cannot rule out that they are genuine TSSs. In our 5′-RACE experiments, a single 5′-end was mapped inside of exon 2, 66 nt downstream from the first nucleotide of transcript RNASEQT00000061379 (TSS3 in Figure 6A, see also Figure S6B for details). In this case, it is likely that the 5′-RACE product represents the truncated form transcribed from TSS3.

RT-PCR analysis of *fbrsl1* expression during zebrafish development revealed that both isoforms are maternally supplied (Figure 6C). Isoforms *fbrsl1-i1* (TSS1) and *fbrsl1-i2* (TSS4) are detected again from 9 to 6 hr, respectively (weak signal at 9 hr for TSS1, Figure 6C). Direct sequencing of PCR products revealed coamplification of transcripts with and without exons 4 or 17 (skipped exons), and coamplification of transcripts with mutually exclusive exons 9a and 9b.

### Expression of the fbrsl1 gene during zebrafish development

We conducted WISH to examine the spatial expression of the *fbrsl1* gene during zebrafish development. Since we identified two populations of *fbrsl1* isoforms, *fbrsl1-i1* and *fbrsl1-i2*, that are generated from TSS1 and TSS4, respectively, we asked if these isoforms are expressed differentially during development. We used a similar approach as for *auts2a* isoforms. We designed two isoform-specific riboprobes that recognize alternative mutually exclusive exons 1A and 1B in *fbrsl1* transcripts (Figure S8A). Analysis of *in situ* hybridization data showed that although expression was quite similar between the two probes, the isoform transcribed from TSS4 was expressed in hypothalamus and ventral diencephalon, which was not detected for the TSS1 probe, and more strongly in the telencephalon (Figure S8B). For further analysis of *fbrsl1* expression during development, we used a probe designed against full-length isoform *fbrsl1-i2b* containing exon 9b (for probe details see *Materials and Methods*).

**Figure 7:**
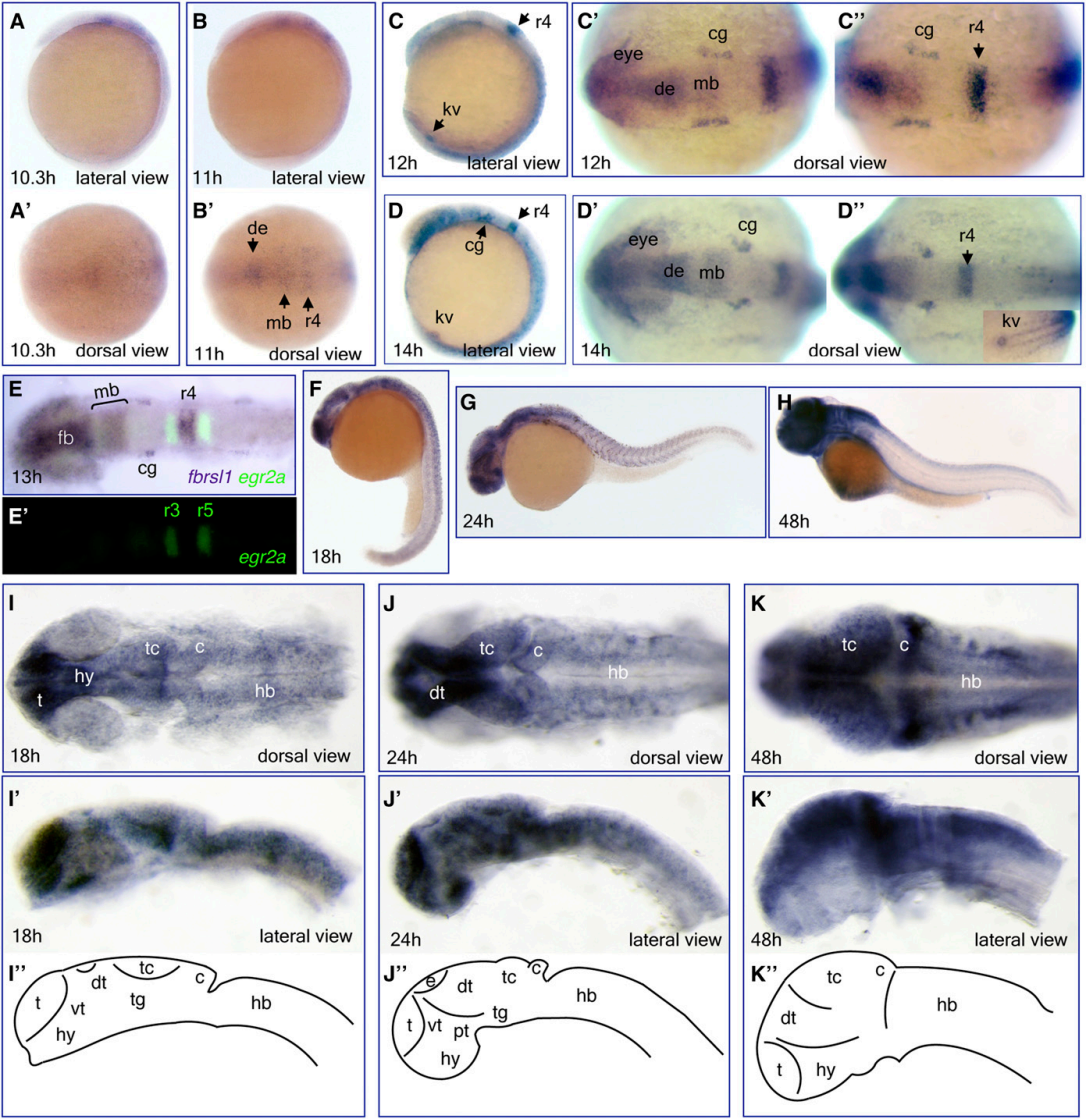
Expression of *fbrsl1* mRNA during zebrafish development. Whole-mount *in situ* hybridization analysis of *fbrsl1-i2b* transcript expression in wild-type embryos at different developmental stages: 10.3 hr (A and A’), 11 hr (B and B’), 12 hr (C–C”), 14 hr (D–D”), 18 hr (F, I, and I’), 24 hr (G, J, and J”), and 48 hr (H, K, and K’). (E and E’) Double *in situ* hybridization with *egr2a* (*krox20*) as a second probe. (I, I’–K, and K’). Flat mount prep of embryonic brain with schematic presentation of brain subdivision at these developmental stages (I”–K”). Eyes were removed in (I’–K’). c, cerebellum; cg, cranial ganglia; de, diencephalon; dt, dorsal thalamus; e, epiphysis; fb, forebrain; hb, hindbrain; hy, hypothalamus; kv, Kupffer’s vesicle; mb, midbrain; pt, posterior tuberculum; r, rhombomere; t, telencephalon; tc, tectum; tg, tegmentum; vt, ventral thalamus.

Despite the detection of the *fbrsl1-i2* transcript at 6 hr by RT-PCR (TSS4 in Figure 6C), weak expression in the neural plate becomes evident only at the 1-somite stage (10.3 hr) (Figure 7, A and A’). At the 3-somite stage (11 hr), *fbrsl1-i2b* is expressed in the presumptive diencephalon, midbrain, and rhombomere 4 (Figure 7, B and B’). During neural keel formation (12–14 hr), *fbrsl1-i2b* expression continues in the forebrain, midbrain, and rhombomere 4. Expression in rhombomere 4 was confirmed using double *in situ* hybridization with *egr2a* (*krox20*) as a second probe to mark rhombomeres 3 and 5 (Figure 7, E and E’). *Fbrsl1-i2b* is also expressed in the spinal cord, cranial ganglia, and Kupffer’s vesicle (Figure 7, C–D”). At 18 hr, expression in the brain is broad with the strongest signals in the telencephalon and hypothalamus (Figure 7, I–I”), and expression continues in somites (Figure 7F). At 24 hr, *fbrsl1-i2b* is strongly expressed in the telencephalon, hypothalamus, dorsal thalamus (Figure 7, J–J”), and somites (Figure 7G). At 48 hr, *fbrsl1-i2b* is strongly expressed in the dorsal forebrain, optic tectum, cerebellum, dorsal hindbrain, and spinal cord (Figure 7, H and K–K”).

**Figure 8:**
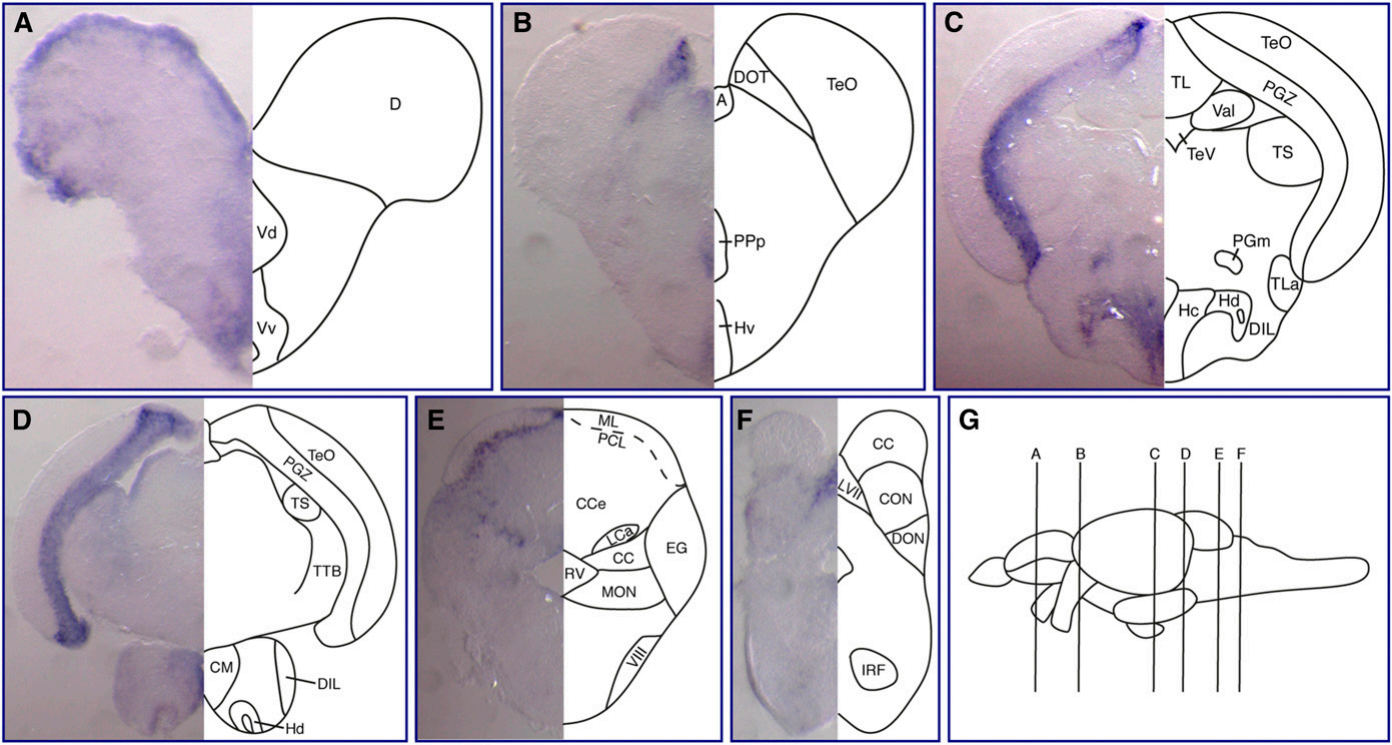
Expression of *fbrsl1* mRNA in juvenile brain. (A–F) Transverse sections at the levels indicated by the vertical lines in (G) show *fbrsl1-i2b* mRNA expression in different regions of the juvenile brain: telencephalon (A), diencephalon and midbrain (B and C), and hindbrain (D–F). Abbreviations used to label areas in brain sections can be found in Table S1.

We also performed *in situ* hybridization analysis of *fbrsl1-i2b* expression in the juvenile zebrafish brain (Figure 8). In the forebrain, expression of *fbrsl1-i2b* is detected in the dorsal telencephalic area (D), the ventral nucleus of ventral telencephalic area (Vv), the ventral (Hv), caudal (Hc), and dorsal (Hd) zones of the periventricular hypothalamus, the mammillary body (CM), and the posterior part of the parvocellular preoptic nucleus (PPp) (Figure 8, A–D). In the midbrain, *fbrsl1-i2b* is strongly expressed in the PGZ and also detected in the medial preglomerular nucleus (PGm) and dorsomedial optical tract (DOT) (Figure 8, B–D). In the cerebellum, *fbrsl1-i2b* is expressed in the PCL, similar to *auts2a*, and in the LCa (Figure 8E). In the hindbrain, *fbrsl1-i2b* is expressed in the LVII and CON (Figure 8F).

### In silico identification of transcripts in the fbrs gene locus and expression of fbrs during development

**Figure 9:**
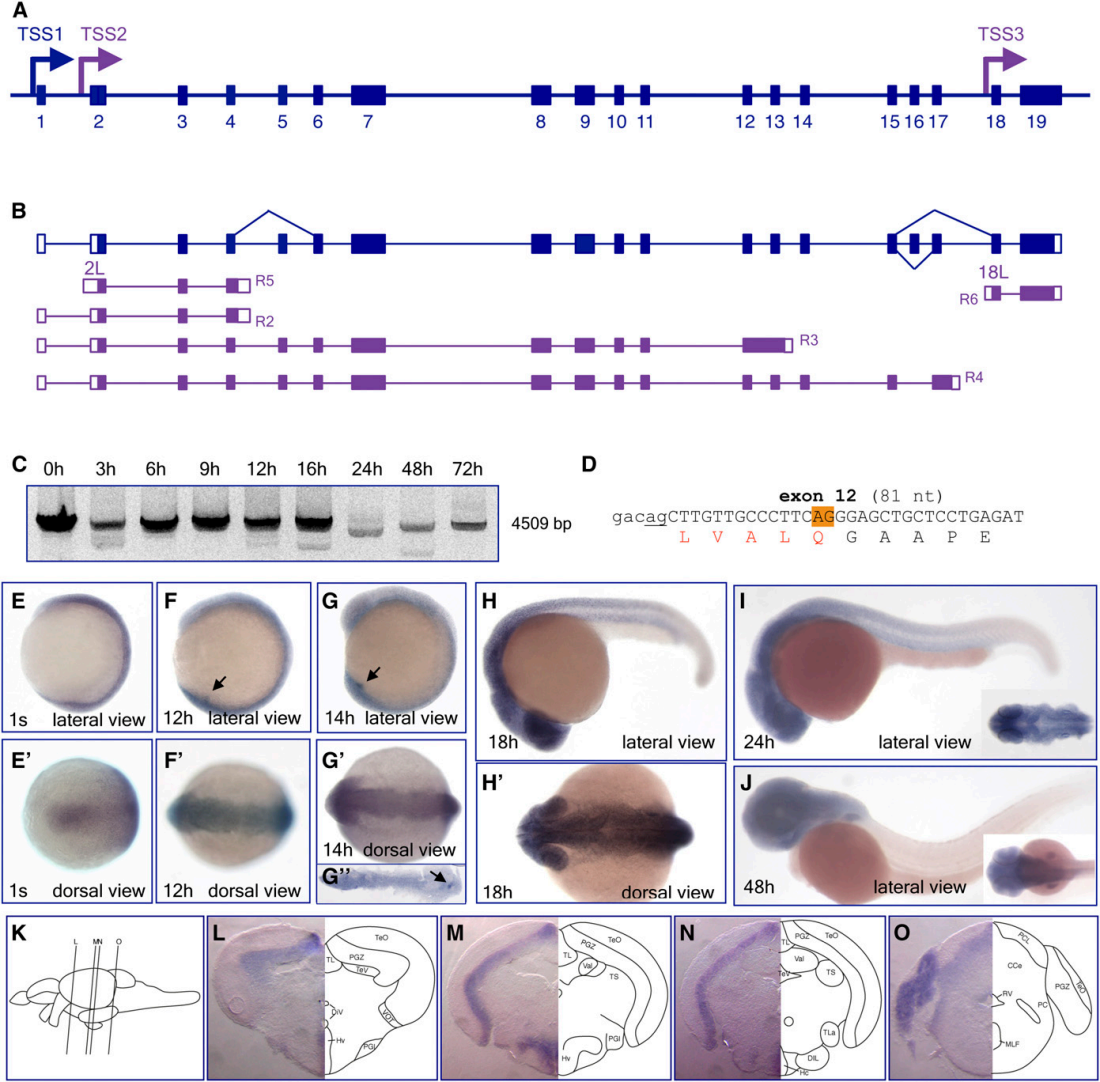
Gene structure, transcript complexity, and expression of the *fbrs* gene during zebrafish development and in the juvenile brain. (A) Schematic presentation of the 20.5 kb long *fbrs* genomic region on chromosome 12 (not to scale). Exons are shown as bars. Arrows show TSSs identified either in this study (blue) or annotated based on RNASeq data (purple). (B) Schematic presentation of structure of *fbrs* transcript identified in this study. Structures of RNASeq transcripts transcribed from alternative TSSs are shown in purple. RNASeq transcript IDs are shown in Table S5. Noncoding and coding exons are depicted as open and filled bars, respectively. (C) RT-PCR analysis of *fbrs* expression during zebrafish development. Primers amplifying the full-length transcripts were used for analysis. The second faint bands are reverse transcriptase template switching artifacts. (D) Partial DNA sequence of alternatively spliced exon 12. Position of alternative 3′ acceptor splice site is highlighted in orange. Constitutive splice site is underlined. Deleted amino acids are shown in red. (E–J) Whole mount *in situ* hybridization analysis of *fbrs* transcript expression in wild-type embryos at different developmental stages, from 1-somite (10.3 hr) to long-pec (48 hr) stages. Arrows point to expression in Kupffer’s vesicle. (L–O) Expression of *fbrs* mRNA in juvenile brain. Transverse sections were made at the levels indicated by the vertical lines in (K). Abbreviations used to label areas in brain sections can be found in Table S1. ID, identifier; RNASeq, RNA sequencing; RT-PCR, reverse transcription-polymerase chain reaction; TSS, transcription start site.

The current RefSeq *fbrs* gene model (NCBI Gene ID: 100535921) defines 19 exons present in transcript XM_003199613 (Figure 9A). Three RefSeq predicted transcripts, XM_005156304, XM_005156306, and XM_017358534, represent alternatively spliced mRNAs that are transcribed from the same TSS1 (Figure 9B). Alternative splicing occurs at the 3′ acceptor splice site of exon 12 (XM_005156304), which leads to the exclusion of 15 nt from the spliced mRNA and results in an in-frame deletion of five amino acids in the protein (Figure 9D). Skipped exons are found in transcripts XM_005156306 (exons 16 and 17 are spliced out together), XM_017358534 (skipped exon 16), and in Ensembl transcript ENSDART00000153054 (skipped exon 5). Transcript ENSDART00000153054 also has an alternatively spliced exon 12 similar to that in XM_005156304.

We performed 5′-RACE analysis with gene-specific primers designed against exon 2. We could identify only a single TSS, which was mapped 55 nt upstream from the first nucleotide of RefSeq transcripts (see Figure S9). Using a forward primer, designed in close proximity to TSS1, we isolated cDNA corresponding to XM_017358534. From RNASeq data, two additional alternative first exons, representing 5′ extensions of exon 2 (2L, TSS2) and exon 18 (18L, TSS3), were predicted (Figure 9B and Figure S9). Analysis of RNASeq data also revealed transcription from TSS1 that generates isoforms with alternative 3′-ends (see Table S5).

RT-PCR and *in situ* analysis revealed that *fbrs* mRNA is maternally supplied and ubiquitously expressed in neural tissues during development (Figure 9, C and E–J). It is also expressed in Kupffer’s vesicle (Figure 9, F, G, and G”) and later in the eyes and somites (Figure 9, H–J). In the juvenile brain, expression of *fbrs* is detected in the Hv, Hc, and Hd zones of the periventricular hypothalamus, the PGZ, and the granular layer of the CCe (Figure 9, K–O).

## Discussion

Eukaryotes employ a range of mechanisms to generate multiple mRNA isoforms. These mechanisms include usage of alternative TSSs, alternative splice sites, and polyadenylation. Here, we show that, in zebrafish, the *auts2* gene family has four paralogs: *auts2a*, *auts2b*, *fbrsl1*, and *fbrs*. All four paralogs exhibit multiple TSSs and alternative splicing, with *auts2a* and *fbrsl1* giving rise to more mRNA diversity than *auts2b* or *fbrs*. However, whether all mRNA isoforms are translated into proteins cannot be easily determined and the interesting question is whether there is a biological function underlying such complexity. Our study was limited to the usage of 5′-RACE analysis followed by RT-PCR and cloning. We have not examined the presence of alternative polyadenylation sites, although available RNASeq data support the presence of RNAs with alternative 3′-ends, with some of them encoding short polypeptides or representing ncRNAs.

### Transcriptional complexity in the auts2a gene locus

Among *auts2* paralogs, the highest level of transcriptional complexity was found in *auts2a*. Here, the complexity is achieved mainly through the usage of alternative promoters rather than through alternative splicing. In this case, transcription from alternative promoters generates *auts2a* mRNAs that differ in 5′-UTRs and encode the N-terminally truncated protein isoforms, while the C-terminal portion of Auts2a protein remains the same. Differences in 5′-UTRs may have an impact on translation efficiency since cap-dependent ribosomal scanning is severely hampered in 5′-UTRs containing upstream AUGs (uAUGs), uORFs, and secondary structures (Araujo *et al.* 2012).

The N-terminally truncated protein isoforms may differ in intracellular localization and/or trafficking. In the human AUTS2 protein, several regions were predicted that may be functionally important: the putative NLS in the N-terminal part, two proline-rich regions (PR1 and PR2), and the PY motif in the middle of the protein (Sultana *et al.* 2002; see Figure S2 for zebrafish Auts2a protein annotation). The relevance of the predicted NLS is not clear, since an AUTS2 protein isoform lacking NLS was localized exclusively in the nucleus (Hori *et al.* 2014). In our experiments, we also observed nuclear localization after transfection of the HEK293T cell line with either the N-terminal end of Auts2a or an Auts2a-i3 isoform that lacks 467 N-terminal amino acids that are present in the long protein isoform (data not shown). It is clear that Auts2 protein may contain another NLS, different from the one predicted in the N-terminal part. Alternatively, the nuclear localization of Auts2 protein might result from its interaction with another nuclear protein.

The PR1 region was shown to be important for the regulation of actin remodelling, and for neuronal migration and neuritogenesis in particular (Hori *et al.* 2014). The C-terminal portion of AUTS2, comprising the Auts2 family domain and His repeats, is important for mediating transcriptional activation (Gao *et al.* 2014) and deletion of this part in the human causes severe phenotypes (Beunders *et al.* 2015). His repeats have been shown to be associated with protein localization at nuclear speckles (Salichs *et al.* 2009). The zebrafish Auts2a protein does not contain His repeats at the C-terminal end.

Splicing of *auts2a* pre-mRNA at tandem splice acceptors with a NAGNAG motif leads to very minor changes in protein sequence (deletion of a single amino acid). It has been proposed that splicing between the intron-proximal and intron-distal AG is achieved by a competition mechanism (Hiller *et al.* 2004). In many cases, the selection of such AGs can be highly regulated under specific spatiotemporal conditions or external stimuli. Tandem splicing of mRNAs can lead to the production of functionally different proteins, for example, the *SCN5A* (Makielski *et al.* 2003) and *pou5f3* genes (formerly *pou2*) (Takeda *et al.* 1994). In the first example, cells expressing voltage-dependent sodium channel α-subunit protein SCN5A, which contains Q1077, showed a reduced inward sodium current in comparison to SCN5A variants lacking Q1077 (Makielski *et al.* 2003). In the second example, the usage of either the distal or proximal 3′ splice acceptor sites leads to the generation of two proteins that possess distinct functions; one is a transcription factor, while the other is a non-DNA-binding protein (Takeda *et al.* 1994). In the case of *auts2a*, alternative splicing at these particular exons is evolutionarily conserved; the positions and identity of deleted amino acids are conserved among either all vertebrates (exons 3 and 8) or only among ray-finned fish, sharks, and coelacanth (exon 15), supporting the functional significance of such minor changes in protein sequence. Splicing at an alternative 5′ donor site of exon 9 leading to an in-frame deletion of seven amino acids is highly conserved among jawed vertebrates. Moreover, splicing at this exon is also conserved between *auts2a* and *fbrsl1* genes: identical amino acids that are present in the full-length Fbrsl1 protein are also removed during alternative splicing of the *fbrsl1* pre-mRNA (Figure 1D and Figure 6D).

### Transcriptional complexity in the fbrsl1 gene locus

Transcriptional complexity in the *fbrsl1* gene is achieved through the usage of both alternative promoters and alternative splicing. Three modes of alternative splicing were found in the *fbrsl1* gene: exon skipping (exon 17), splicing at alternative 5′ donor splice sites of exons 9b and 16, and mutually exclusive exons 9a and 9b. Interestingly, from RNASeq data, two potential TSSs are annotated inside of exons 9a and 9b. Although the importance of exonic promoters is highly speculative, exonic TSSs might have some relationship to so-called exonic splicing enhancers influencing the recruitment of SR proteins (Carninci *et al.* 2006). Some have speculated that RNAs generated from these TSSs may regulate the decision of which exon will be used in the protein-coding transcript. Differential usage of these mutually exclusive exons leads to protein isoforms that differ substantially in sequence. Amino acid sequence coded by exon 9b is highly conserved even between paralogs. Exclusion of exon 9b may have a drastic effect on protein function. Currently, there is no experimental evidence for a functional role of FBRSL1, except that in RNA-bound proteome analysis FBRSL1 was identified as a candidate RNA-binding protein (Baltz *et al.* 2012). However, the identity of the particular FBRSL1 protein isoform involved was not reported.

### Implications of transcriptional complexity for disease

Although transcriptional complexity allows greater flexibility and control in complex systems, it is more likely to be misregulated, particularly in systems that depend heavily on alternative splicing. Aberrant promoter usage has been associated with several human cancers including colon cancer, ovarian cancer, and neuroblastomas (Landry *et al.* 2003; Davuluri *et al.* 2008), suggesting that genes with alternative promoters are more likely to be associated with disease (Davuluri *et al.* 2008; Liu 2010). *BDNF* utilizes multiple promoters in a tissue-specific manner, and promoter usage is altered after kainite-induced seizures (Timmusk *et al.* 1993). A SNP in the promoter region of the 5-HT2A receptor affects promoter activity and is associated with psychiatric disorders (Parsons *et al.* 2004). Several SNPs, identified within the promoter region of the *Kalirin* gene are associated with coronary artery disease (Wang *et al.* 2007; Horne *et al.* 2009; Boroumand *et al.* 2014). In this context, the transcriptional complexity in *auts2* family genes that we describe here provides an exciting opportunity to understand how misregulation of transcription at these loci leads to the disease conditions that they are associated with.

### Expression patterns of auts2 paralogs

Differential patterns of gene expression among paralogs is widely believed to play a prominent role in morphological diversification. Duplicated genes are considered to diverge through neofunctionalization (Ohno 1970) and/or subfunctionalization (Lynch and Force 2000), but both processes can occur through evolution of the CDSs and/or the regulatory sequences, giving distinct and/or novel sites of expression.

Previously, it was shown that *auts2a* is ubiquitously expressed in the central nervous system beginning from 24 hr (Oksenberg *et al.* 2013). Our data show that, already at very early stages, the expression of *auts2a* becomes restricted to the neural plate. Except for the *fbrs* gene, which is expressed ubiquitously through development, other *auts2* paralogs—*auts2a*, *auts2b*, and *fbrsl1*—show distinct expression patterns, particularly in the hindbrain, suggesting their role in patterning the hindbrain. For example, during neural keel formation, expression of *fbrsl1* in the hindbrain is mainly detected in rhombomere 4, while *auts2a* is expressed in rhombomeres 1, 2, and 4, and *auts2b* is expressed more broadly, with the strongest expression in rhombomere 2 (see Figure S10). Expression of *fbrsl1* and *fbrs* genes in the Kupffer’s vesicle suggests their potential involvement in the establishment or maintenance of left–right asymmetry.

Analysis of expression of *auts2* paralogs in juvenile brains revealed the presence of these transcripts in proliferation zones, suggesting their role in adult neurogenesis. In contrast to mammalian brains, teleostean brains have a tremendous number of proliferation zones. Many of these zones are found at or near the surfaces of ventricles (Zupanc *et al.* 2005). Three paralogs—*auts2a*, *fbrsl1* and *fbrs*—are localized in the PGZ of the optic tectum, a site known for mitotic activity in the midbrain. In the cerebellum, proliferation zones are located in regions distant from any ventricle. Quantitative analysis has shown that the majority of the new brain cells are generated in the cerebellum; the proliferation zones are located in specific areas within the molecular layers of the cerebellar corpus and the valvula cerebelli (Zupanc and Horschke 1995; Hinsch and Zupanc 2007). *Auts2* paralogs are localized either in the granular layer (*auts2b* and *fbrs*) or Purkinje cell layer (*auts2a* and *fbrsl1*), sites where mitotic activity is minimal. Interestingly two *auts2* paralogs, *auts2b* and *fbrsl1*, are also expressed in the caudal lobe of the cerebellum, a granular layer of the eminentia granularis, the other site of mitotic activity in the cerebellum (Zupanc and Horschke 1995; Hinsch and Zupanc 2007).

### Evolution of the auts2 gene locus

To our surprise, only cave fish and zebrafish possess a full-length copy of the *auts2b* gene. The other teleosts (where sequenced genomes are available) retain a highly reduced copy of the *auts2b* gene. Moreover, in the cave fish genome we could not find an *auts2a* gene and it is currently unclear if the absence of *auts2a* gene in the cave fish genome is simply due to incomplete sequencing and assembly, or it has indeed evolved beyond recognition. Phylogenetically, cave fish and zebrafish belong to the *Otomorpha* group. *Otomorpha* and *Euteleosteomorpha* (all other teleosts with sequenced genomes) split ∼245 MYA (Broughton *et al.* 2013). The most common fate of duplicated genes is that while one of the duplicated genes continues to be under selective pressure and retains the ancestral function, the other gene diverges and becomes nonfunctional through the accumulation of deleterious mutations (Langham *et al.* 2004). Less frequently, both genes are retained, which is the case for the *auts2a* and *auts2b* genes in the zebrafish genome. Although in other teleost genomes *auts2b* has evolved almost beyond recognition, transcription from this genomic locus could still be detected, as suggested by RNASeq data derived from amazon molly, tilapia, and platyfish transcriptomes. It will be interesting to examine how these transcripts are spatially expressed.

### Conclusions

Taken together, our results show the existence of multiple *auts2* paralogs in zebrafish and the usage of alternative promoters and alternative splice sites for generating huge diversity in mRNA transcripts. The expression of these gene products is also tightly regulated developmentally and across multiple brain regions. Such complexity in regulation of these loci is bound to have significant functional roles and our future studies will be aimed at deciphering them.

## Supplementary Material

Supplemental material is available online at www.g3journal.org/lookup/suppl/doi:10.1534/g3.117.042622/-/DC1.
