# Supplementary figures and images for: Transcriptional Complexity and Distinct Expression Patterns of auts2 Paralogs in Danio rerio
Source: G3 (Bethesda). 2017 Jun 16;7(8):2577–93. doi: 10.1534/g3.117.042622 (PMC5555464; doi:10.1534/g3.117.042622)

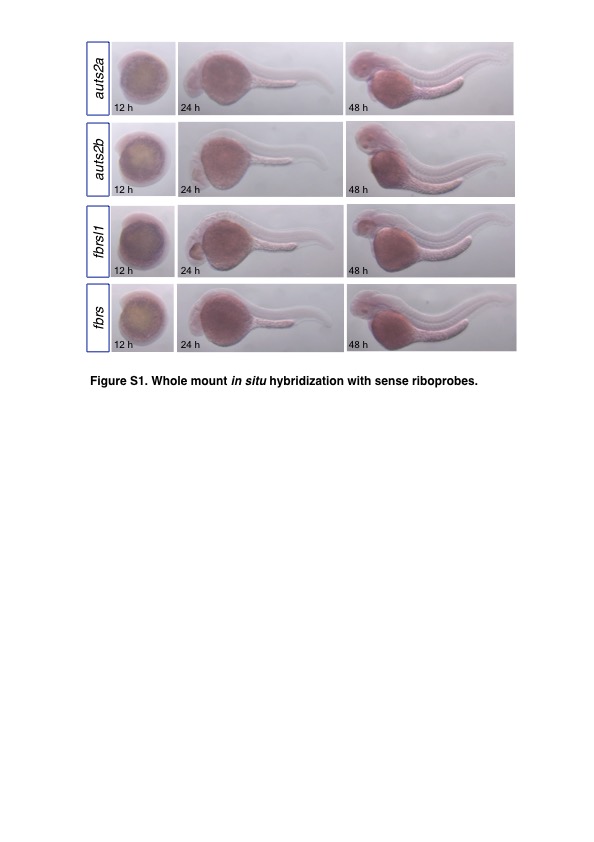

Supplement: Supplementary file 1 [file 2577FigureS1.jpg]

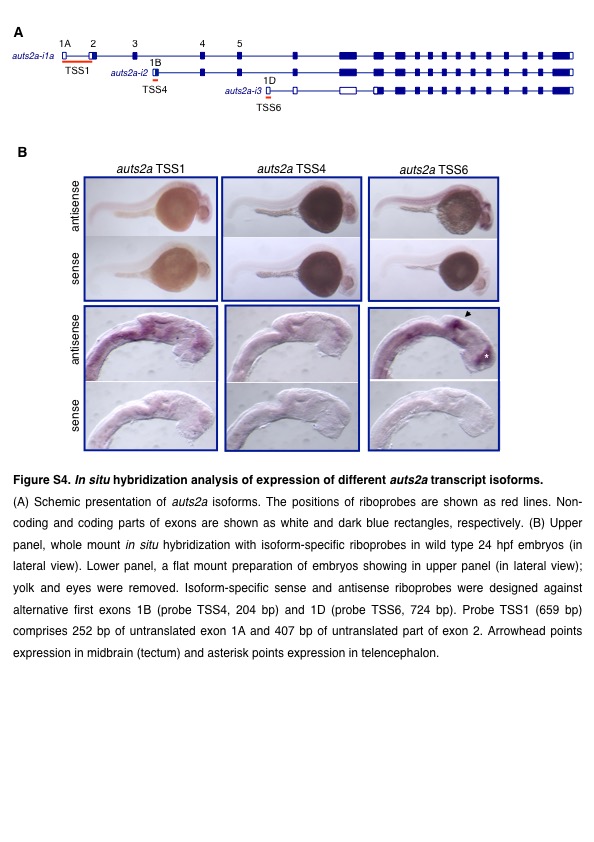

Supplement: Supplementary file 4 [file 2577FigureS4.jpg]

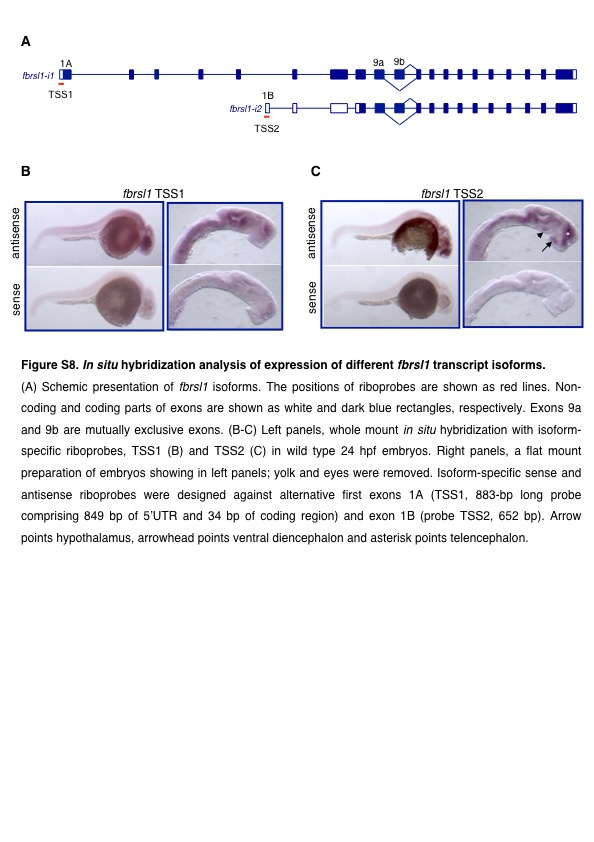

Supplement: Supplementary file 8 [file 2577FigureS8.jpg]

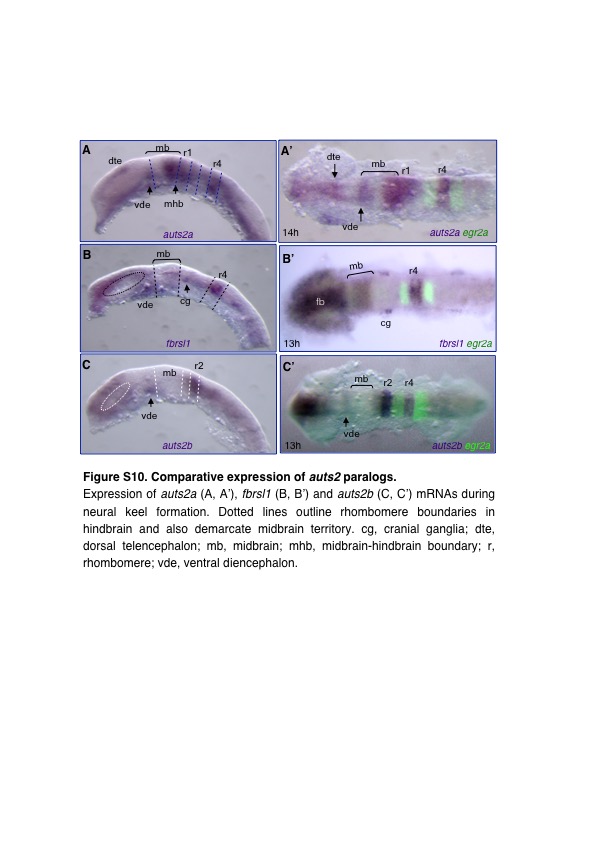

Supplement: Supplementary file 10 [file 2577FigureS10.jpg]
