# Supplementary material for: Transcriptional Complexity and Distinct Expression Patterns of auts2 Paralogs in Danio rerio
Source: G3 (Bethesda). 2017 Jun 16;7(8):2577–93. doi: 10.1534/g3.117.042622 (PMC5555464; doi:10.1534/g3.117.042622)
Supplement: Supplementary file 2 [file 2577FigureS2.pptx]

## Slide 1
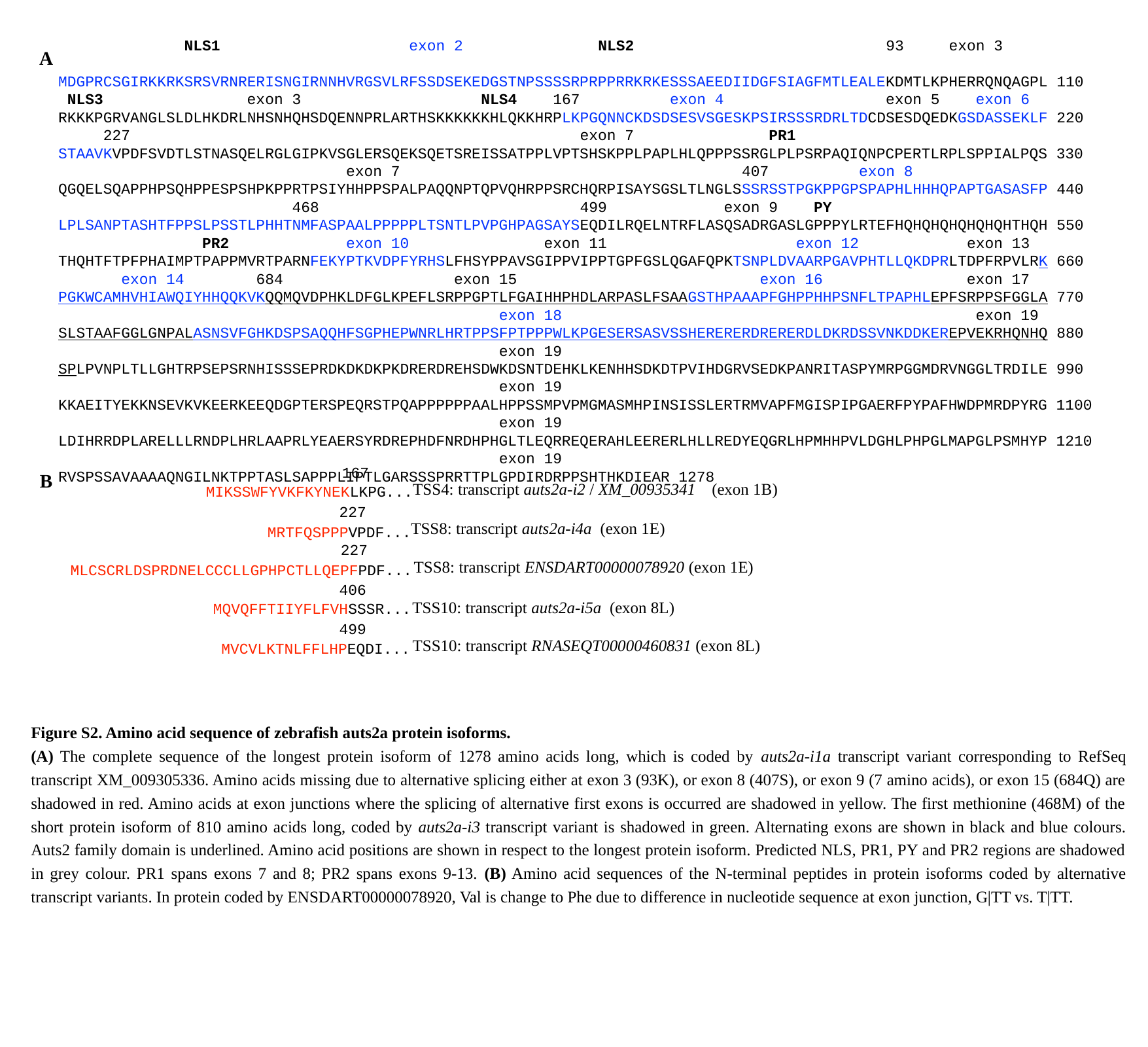

A
 NLS1 exon 2 NLS2 93 exon 3
MDGPRCSGIRKKRKSRSVRNRERISNGIRNNHVRGSVLRFSSDSEKEDGSTNPSSSSRPRPPRRKRKESSSAEEDIIDGFSIAGFMTLEALEKDMTLKPHERRQNQAGPL 110
 NLS3 exon 3 NLS4 167 exon 4 exon 5 exon 6
RKKKPGRVANGLSLDLHKDRLNHSNHQHSDQENNPRLARTHSKKKKKKHLQKKHRPLKPGQNNCKDSDSESVSGESKPSIRSSSRDRLTDCDSESDQEDKGSDASSEKLF 220
 227 exon 7 PR1
STAAVKVPDFSVDTLSTNASQELRGLGIPKVSGLERSQEKSQETSREISSATPPLVPTSHSKPPLPAPLHLQPPPSSRGLPLPSRPAQIQNPCPERTLRPLSPPIALPQS 330
 exon 7 407 exon 8
QGQELSQAPPHPSQHPPESPSHPKPPRTPSIYHHPPSPALPAQQNPTQPVQHRPPSRCHQRPISAYSGSLTLNGLSSSRSSTPGKPPGPSPAPHLHHHQPAPTGASASFP 440
 468 499 exon 9 PY
LPLSANPTASHTFPPSLPSSTLPHHTNMFASPAALPPPPPLTSNTLPVPGHPAGSAYSEQDILRQELNTRFLASQSADRGASLGPPPYLRTEFHQHQHQHQHQHQHTHQH 550
 PR2 exon 10 exon 11 exon 12 exon 13
THQHTFTPFPHAIMPTPAPPMVRTPARNFEKYPTKVDPFYRHSLFHSYPPAVSGIPPVIPPTGPFGSLQGAFQPKTSNPLDVAARPGAVPHTLLQKDPRLTDPFRPVLRK 660
 exon 14 684 exon 15 exon 16 exon 17
PGKWCAMHVHIAWQIYHHQQKVKQQMQVDPHKLDFGLKPEFLSRPPGPTLFGAIHHPHDLARPASLFSAAGSTHPAAAPFGHPPHHPSNFLTPAPHLEPFSRPPSFGGLA 770
 exon 18 exon 19
SLSTAAFGGLGNPALASNSVFGHKDSPSAQQHFSGPHEPWNRLHRTPPSFPTPPPWLKPGESERSASVSSHERERERDRERERDLDKRDSSVNKDDKEREPVEKRHQNHQ 880
 exon 19
SPLPVNPLTLLGHTRPSEPSRNHISSSEPRDKDKDKPKDRERDREHSDWKDSNTDEHKLKENHHSDKDTPVIHDGRVSEDKPANRITASPYMRPGGMDRVNGGLTRDILE 990
 exon 19
KKAEITYEKKNSEVKVKEERKEEQDGPTERSPEQRSTPQAPPPPPPAALHPPSSMPVPMGMASMHPINSISSLERTRMVAPFMGISPIPGAERFPYPAFHWDPMRDPYRG 1100
 exon 19
LDIHRRDPLARELLLRNDPLHRLAAPRLYEAERSYRDREPHDFNRDHPHGLTLEQRREQERAHLEERERLHLLREDYEQGRLHPMHHPVLDGHLPHPGLMAPGLPSMHYP 1210
 exon 19
RVSPSSAVAAAAQNGILNKTPPTASLSAPPPLIPTLGARSSSPRRTTPLGPDIRDRPPSHTHKDIEAR 1278
167
B
TSS4: transcript auts2a-i2 / XM_00935341 (exon 1B)
MIKSSWFYVKFKYNEKLKPG...
227
TSS8: transcript auts2a-i4a (exon 1E)
MRTFQSPPPVPDF...
227
TSS8: transcript ENSDART00000078920 (exon 1E)
MLCSCRLDSPRDNELCCCLLGPHPCTLLQEPFPDF...
406
TSS10: transcript auts2a-i5a (exon 8L)
MQVQFFTIIYFLFVHSSSR...
499
TSS10: transcript RNASEQT00000460831 (exon 8L)
MVCVLKTNLFFLHPEQDI...
Figure S2. Amino acid sequence of zebrafish auts2a protein isoforms.
(A) The complete sequence of the longest protein isoform of 1278 amino acids long, which is coded by auts2a-i1a transcript variant corresponding to RefSeq transcript XM_009305336. Amino acids missing due to alternative splicing either at exon 3 (93K), or exon 8 (407S), or exon 9 (7 amino acids), or exon 15 (684Q) are shadowed in red. Amino acids at exon junctions where the splicing of alternative first exons is occurred are shadowed in yellow. The first methionine (468M) of the short protein isoform of 810 amino acids long, coded by auts2a-i3 transcript variant is shadowed in green. Alternating exons are shown in black and blue colours. Auts2 family domain is underlined. Amino acid positions are shown in respect to the longest protein isoform. Predicted NLS, PR1, PY and PR2 regions are shadowed in grey colour. PR1 spans exons 7 and 8; PR2 spans exons 9-13. (B) Amino acid sequences of the N-terminal peptides in protein isoforms coded by alternative transcript variants. In protein coded by ENSDART00000078920, Val is change to Phe due to difference in nucleotide sequence at exon junction, G|TT vs. T|TT.
