# Supplementary material for: Transcriptional Complexity and Distinct Expression Patterns of auts2 Paralogs in Danio rerio
Source: G3 (Bethesda). 2017 Jun 16;7(8):2577–93. doi: 10.1534/g3.117.042622 (PMC5555464; doi:10.1534/g3.117.042622)
Supplement: Supplementary file 3 [file 2577FigureS3.docx]

**A)** **exon 1A (TSS1)**

5’...accgagtcctcag**tatataaaaa**gcgcagcactatatgcgctgaggaagcggattcctgtaaagcggcactg

CGGCAGTGCTGTATCAATGCGCTCGGTCCCCGCTCGCTCCGGGATCCTGAAGCACGGACTCGCGGAGAGGAGCAGCGTCGCTTCCTTGCAGGATTTATTTGTTTGTGGAAAAATTTATCTTTTGTTTTCCGTTCAAGAGCAGCGCGCTTTCCGAATGCCGAAGACTCGTTCGGACGACCCCCCTCCACTCCCCAGGCTTTGCATGTGTTGCTTTTGTCCTGCAGGAGAGAAGGCAGGCTTTGTGTTTTGGTGGATCTAGAGCTGAAGGAGGCAGTGCCAGGCGCTGCTGCTGCTGCTGCTGCTGTGGGCTGGACCGGCCACGgtgcgtgagatttactgacggagagagaaaagaaagagagagagagacaaaaaaggactcatggaagccagcgagagtgaagggagggttggcagggagtgtaaatatatcaaaaccatttatgaatttgag**gt**gtgtgtatgtgtgtgt...3’

The first nucleotides in RNASeq transcript R1, EST CK397258, RefSeq transcript XM_009305336 and 5’-RACE products are shadowed in colour code. Predicted TATA-box is highlighted in bold. Alternatively used 5’ donor splice site is shown in bold red (found in transcript variant *auts2a-i1d*). Position of forward primer used to clone cDNA is underlined.

B) exon 2L (TSS2) is 5’ extension of exon 2

5’...tcagtcgctttattcataTTCCTGCATTATGTATTTCTATGTCTAGCTAAGGGTGGTGATTCTGAGTAAGCTCTTACAAAGAACATTTCAAACGTCTGTTCTCTATCTCTCCCTTTGACTTGTAGGATGGGAAGGTGTCAGACTGCACTTAAACAGAGTGTTGGGTCCGTGAGGACAGCCATGTGGAGACCTGTTACAGCTCCATTGGAAGACACTTCAAGTGATCTGCCAGTTTGTTTCCCTTAAAACGTGGGCTGAATTTGGAACATTGGATACTTTCCAATTTGGCAGGATTCAACTCGGGATTTCTCTGCTTGTGGATATATGCATTTATCCTTCTGGAATCTCATGTGGGTATCGCACTTCAAGCTAAGTCACATTACATCAGCTGATTCGGGAATTTGGATAATTGTGCATCTTTTGCTGGGGCACAGTCGTTGAGCTTTTCATTGCAGGTGTTTGTTGGGAATCGAGTTCTGCTTTCACCTCTGAGAGCCGCCTTCTAGGAAGTGTTCGGTCGAGTGTGTCATCTGGTGAAATGGATGGCCCGCGGTGCAGCGGGATCCGGAAAAAGCGCAAGTCTCGGTCGGTGCGCAACCGGGAGCGCATATCTAACGGGATCAGGAATAACCATGTCCGGGGCTCCGTGCTTCGCTTCTCCTCGGATTCTGAGAAGGAAGACGGCAGCACCAACCCCTCCTCTTCCTCACGACCCAGGCCACCGAGGAGGAAGAGGAAGGAGTCTTCATCTGCCGAAGAGGACATCATTGATGGATTCTCAATTGCGGGATTTATGACGTTGGAAGCTCTTGAGgtaagaagcc...3’

The first nucleotides in RNASeq transcripts R3 (T) and R2 (C), and in 5’-RACE product (TSS3) are shadowed in colour code. Translation start codon of the long isoform *auts2a-i1* is shown in bold blue.

C) exon 1B (TSS4)

5’...tagagaacgaGACTGAAAGACGCATAAGCAGACAGAGGGATTGAGCTCTGCTGGAGAAGACCGTCTGCATTCAGATCTCCATCTTCTGGCGTTGTGCACTGACACCGGACCTCTCACCCTGTCTGACCTGTCCTGACCTGTCCCGCTACCCGCAGGCGCACTACACGCTCCGTGCCGATAGACGACACC**ATG**ATTAAGTCCAGCTGGTTCTATGTCAAGTTCAAATATAACGAGAAGgtgagtcact...3’

The first nucleotides in RNASeq transcript R4, 5’-RACE product and RefSeq transcript XM_009305341 are shadowed in colour code. Translation start codon of alternative isoform *auts2a-i2* is shown in bold blue. Position of forward primer used to clone cDNA is underlined.

D) exon 1C (TSS5)

5’...aatggcagagCTTCAGAAGACGTAAATTGCAGACCTTGACGACAGTTGCTGGGTCCCAGGAGTATGGCATTGTTCTCCAGACTTTACTGGGGCCTCAGATGTTTTGGGATTTTGCGTCCTCCTCCCGTCCCCCCGCCTCCGTCCTTCTCCTCTGTGCTCTGCTCTTGTTCTTTATGATTTGTAGTCTGCTGAAGGACACCTGCTGTGAATCTTTGGTTCTTGCGTGAGGGCTTACCGAATGGAAAACTGCTTTTGATGTAGTTTCGCGAAATTTGATTTCAGTTTCTCACAAATCTGATAGTAAAGGGACAGTTTAATAAAAATGAAATTCAGgtattactt...3’

No RNASeq transcripts were associated with this exon. Position of forward primer used to clone cDNA is underlined.

E) exon 1D (TSS6)

5’...ttattttaaaATCATAATTTTAGGCTGCATTTATATCGATCTAGACTCTCTCTCTGTTTCTCTCTCTCTCTCTCTCTCTCTCCTGTTTGCGCTCTTTTAAGGACTCTGCTCCTAGTAGCTCTAACTGAGCGCCGTAACATTACACGCACACACACGCACGCGCGCGCACGCGCGCGCACGCACTCCCGCACTGTCTGTCCGGTGAAGCTGAGCGGAGGAAGCGCTCTCCGTGTGTGTGCGGCAGAGAGAGGCAGGGGGTCGGAGCTGAATGAAGTCAGGTTAGATTCAGGAAGTGGGAGGACCCGGTCGCGCTGTGCTGAATGAGAGTTTGTGTGGATACAGGCAGCACTTGCGCGAGGAACGGGAGCCAGCGACAGGACACGAGCGTAAAACCGGCGACGTTTTCGAGGAAAGGAAGCGGTGGACGAGGTTTTAGTGCGGCTTTACACCACCCTTTCTGCCTAAAGGAGGAAAATACTGTACGCTTTGTTGGATTTTCTTGCTCGGCACGAGCGCACGGGAGAGAGGCGCGCGCGGATGGGTTGTCACTGAGCGCGCCCCTGACACAAAAGCGCATAGGCGACGGTGTCCGTTAAACGACGGTTGGCGAGGATTTCAAACGACAAATTCGCGCGAACTGCAGGATTTCTTCACTTTTCTGCGCTCTTGGGTACCAGACACATGGCTGCCTTTGTCCGAGAGCCTCTCTCGCTTTGAGCTTTGGATGTTTTTCTACTGCTTCCCTGGGAAAAGGCTTCTTATTTTGCATGGCTGTCATTCTCTCAAAGGGAGCGAGAGAAATTCGCCGCCTGTTTTTGTGGATTACATTGTTTGACAAGCAGAATGTGAAACGGCGTATTTACTAAACCCTCTAAAAGTGATTTCTTGACTTATTTTCCTCCGTGCCAATATCGACACATCAAAAGgtacaccgca...3’

The first nucleotides in RNASeq transcripts R5 (A) and R6 (C), and in 5’-RACE products are shadowed in colour code. Position of forward primer used to clone cDNA is underlined.

**F) exon 6L (TSS7)** is 5’ extension of exon 6 5’...acgctggatgTTTGCACAATAACATAGTAATGTTAATGCGTTCAACCCTGTCTCAAGTGCAGCGTGTGGTTTTGCGCATGGAGCCACAGTGCCATCCAGTGGCCGCTCTGGGAAGCGACTGAATTTCTGCGCTCATTAGTGCTGCGCTACCTTTGATGATGAAGCCCACCTGCTTAGTTGTGAAGCAGATGTACTGACTCTCTCTTTCTCGCTCTCTCTCAGGGCTCAGATGCCAGCTCAGAGAAACTCTTCAGCACAGCTGCAGTTAAAGgtatggtgga...3’

The first nucleotides in RNASeq transcripts R7 (T) and R8 (A). Position of primer used for 5’-RACE is underlined.

**G) exon 1E (TSS8)**

5’...tacttgaacgTGCAAAACAAAGTGGTAGGGAAAGGAAAGGGCTGAGGGGTGGAATTTGGATTGGGCCTTAGTTTGTGTGAACATTCAGGATGTGACTGCAATTGATTAAATAATTGTGAAATATCTGTTGTGTTCGGTGTACTCTCATAAACACATTTTATGTATAAGGTCTCTGAGCTCTGTCTGTGGTTTTCTAGCCGCTGTAT**ATG**CTGTGTTCTTGCAGACTGGACTCACCTCGGGATAATGAGCTGTGCTGCTGTCTGCTGGGACCTCACCCATGCACACTGCTCCAGGAGCCTTGTAGACCTTAATCCTGCTAAAAAAACACATACACATACATAAGCAGTGTGTGACCAG**ATG**AGAACTTTTCAAAGTCCGCCTCCCGgtctgttcta...3’

The first nucleotides in RNASeq transcript R9 and Ensembl transcript ENSDART00000078920. Donor splice site used by RNASeq and Ensembl transcripts are highlighted in red. Translation start codons are highlighted in bold blue. First ATG is a start codon in RNASeq and Ensembl transcripts, second ATG is a start codon in *auts2a-i4a* transcript. Position of forward primer used to clone cDNA is underlined.

**H) exon 7L (TSS9)** is 5’ extension of exon 7

5’...gagctttctaaAAAGTGCTTTGTTGTTCTTTTGTCTCTACAGTTCCAGATTTTAGTGTTGACACACTCTCCACCAATGCCAGCCAGGAGCTGCGAGGCCTGGGCATTCCCAAGGTGTCAGGCCTAGAGCGCAGTCAAGAGAAGAGCCAGGAGACTTCCCGGGAAATCTCCTCGGCCACCCCCCCACTGGTGCCCACTTCCCATTCCAAACCTCCCCTCCCCGCCCCTCTTCATCTTCAGCCACCCCCTTCCAGCAGGGGGTTACCTTTGCCCTCGAGGCCAGCTCAAATCCAGAACCCTTGCCCAGAGCGCACACTAAGGCCACTGTCACCCCCTATTGCTCTGCCTCAATCCCAGGGTCAGGAGCTCTCTCAGGCACCCCCGCATCCGTCTCAGCACCCACCTGAATCACCATCTCACCCAAAGCCACCTCGAACCCCAAGTATCTACCACCACCCCCCATCGCCAGCGCTACCAGCCCAGCAGAACCCTACGCAACCCGTGCAGCACAGGCCACCCTCACGCTGTCACCAGCGTCCAATTTCAGCCTACAGTGGCAGCCTTACGCTCAACGGCCTGAGgtgagtgtgca...3’

The first nucleotide in RNASeq transcript R10.

**I) exon 8L (TSS10)** is 5’ extension of exon 8

5’...cccagtatccCGCAGAGAAGCAGATCTTCCCCTCCCTCCTCTTGCCCTTCTCTGCCGTCCTTTAGATTTGTTGTGTAGTCCACTCAGTGCGGTATATTGCCAGTGGACTGCTGATGATAATTATACTTTGTGTTATTCTGTGTGCGCAGCTTTTAATATGAATATAATTAGCATTTAAATGATTACTCGGCCTGTAAAAGAGCTCTTTGTTAGATTGCGTATAACTCTGATAAATACTTTAGCATGTGTGGCTGGGAAATTCCTCCAGGAATAACACATTCTTTAGTGTATGCACACTTTCCAGGTTTCTGAAACTAAATCCTTTTCTAAGTTTATAGCAGACACTCCCCACTCTTTGAGCAATTTAGTGAACAAAGGAGTTTTGATATTAAAGTTTGGAAAGGGAGTTATTTTTA**ATG**CAGGTGCAGTTTTTCACAATCATTTATTTTCTCTTTGTTCACAGCAGTAGTCGAAGCAGCACTCCGGGGAAGCCTCCGGGCCCATCTCCAGCCCCTCATCTGCACCATCACCAACCTGCCCCCACTGGGGCATCAGCCTCATTCCCCCTGCCCCTGTCAGCGAACCCTACAGCTTCACACACCTTCCCCCCCTCCCTGCCATCCTCCACTCTTCCTCATCACACCAAT**ATG**TTTGCGTCGCCTGCTGCTCTGCCTCCACCTCCTCCTCTCACCTCAAACACTCTGCCGGTCCCCGGACATCCAGCTGGGAGTGCCTACTCAGgtaccagtca...3’

The first nucleotide in RNASeq transcripts R11. Translation start codons are highlighted in bold blue. First ATG is a start codon in *auts2a-i5a* transcript, second ATG is a start codon in *auts2a-i3* isoform, transcribed from TSS6.

**J) exon 13-14 (TSS11)** comprises of exon 13, intron 13 and exon 14

5...cctctctagCTGACAGATCCGTTCCGGCCGGTTCTCAGGGTAAGACTGCTTTATCAGTTATTCGGTTTGTTTCCCCATAGGACTCTGCAGGAAATAGAGTAAAAGCTCATTACAGTTTAATCCCACCTTGCACCGAGCACCTGCCTTCCACTCCGCAGTCTCTTTAATTAGCTGTGCGCTGGGACTCGACAGTCTCATCTCCGTCTATAGGAGACACACTATATGACTCCCTTATCTGAAAACACTTTGTATTGACATTTTGTGTAATTAATTTGCAGCCGTTCCCAAAAATCATTTTAAAGAGTTCAAACATATTCAATTAAATTGGAAAATAATCTTGTCAGATGCACCCAACATTGCTCTATAGGAGTGTTTGCTAGATTTTAGACACAGTTAAACTCATCTGAATTTGCTCATCTGAACTCAGCTTTTCTTTCTGTCTTCACAGAAGCCAGGAAAGTGGTGTGCTATGCATGTTCATATAGCCTGGCAAATTTACCACCACCAACAGAAAGTCAAGgtgagatttg...3’

The first nucleotide in RNASeq transcripts R15, R16 and R17.

**K) exon 16L (TSS12)** is 5’ extension of exon 16

5’...attttactTCTCTCAATATCTATTAAGGAAGTTTTTTAAACCTATTATGATGTAGCTGGGTGACTTGCTCAAAAGTTGTAACCAATTTTATTGTTTTCTGTCTTCCAGGGTCCACGCATCCTGCAGCTGCACCTTTCGGACACCCTCCACATCATCCTAGCAACTTTCTTACTCCAGCACCTCATTTAGgtaaaccta...3’

The first nucleotide in RNASeq transcript R19.

**L) exon 17L (TSS13)** is 5’ extension of exon 17

5’...caattactgaGATGTCCATGTATAGCTGATGTCCATGTAAACGTAGCCATTGATAGCTGTTTCAGAGATTTAATATCTACTAGATCTGAACAGATTAGTGGCCCTGCATGAACACCTCTGGCCTAAGAAATACTAAACACTTAGTGAGAAAACAAGTTGGGTTATCTGTTCATTTGTGAACTAGCTAGCTCTATTGTAAGCATTTGCTGTAATTGTTTGGTGAATTGCTCAGTCAGTTTTCTAATTCTTTGGTTTTGTTTTGACAGAGCCCTTCAGCAGACCGCCTTCATTTGGAGGTCTGGCCTCATTAAGCACTGCAGCCTTCGGTGGTCTGGGAAATCCAGCACTCGgtgagtgtca...3’

The first nucleotide in RNASeq transcripts R20.

Figure S3. Multiple transcription start sites (TSSs) in *auts2a* gene locus.

The first nucleotides (TSSs) annotated in RNASeq, RefSeq, EST, Ensembl and 5’-RACE transcripts are shadowed in green, yellow, pink, blue and red colours, respectively. ID numbers of RNASeq transcripts are provided in Table S2. Exonic and intronic sequences are shown in upper and lower cases, respectively. Constitutive exons are highlighted in grey colour.
