# Supplementary material for: Transcriptional Complexity and Distinct Expression Patterns of auts2 Paralogs in Danio rerio
Source: G3 (Bethesda). 2017 Jun 16;7(8):2577–93. doi: 10.1534/g3.117.042622 (PMC5555464; doi:10.1534/g3.117.042622)
Supplement: Supplementary file 5 [file 2577FigureS5.docx]

**A) exon 1A (TSS1)**

5’...tagagacggaGGAGTGCAGGGAGCTTTCATCTGCCTCCTCCCACAGGGGTGTTGACGCTCCTAATAAATTATGTGATATATCTGTTGTGATAATGCCCCGTCTTGTGATTTATCATCTCACCATGCCCTTTTCCCGCTGACATTCACAGCTCCCAGTCCGACGGGGAGCTCTGCGATTGGTGGATCTCGACAAGAATTCCTCAACATGTATGCGAGGCTTTTATAAAAAGCTGTGACAGGTACGAACGCAACACTTGCATTAAATCATATCATCCTTTTCTCACCCTACGAAAGCACATACCAGCACCGTGGCATTAATTGGAGCTGACGATTTCGCCCAATGAGAGCAAAGTCTCATTTGAGTGTGAGGTGGAAGCATCTCATCGGCATTCTGGATGTGTCGGCTGTCCCCTCTGGTTATTCTGGGCCTGTAATTAAGAGGATGGCCATAATCACTGGACTGTCTGTACGTACGTGTCGAGCAAAGAGGAGGAGAGTGCTGTAATTGTTCTGCTCTCCGGGCCGAGGTGGGAGGCGGTGGGAGTGTCTGCTGAGGGAGGGAGGGATCACAGGAATGTGCTGTTCACTACTGAAGGAGCATTCTTGAGCCGGACGCGCTCTGGAAAGGATCTCTGCATGATCACAGACCTGCTTCGGGTGGATTTGCACTTAATCTGTTGGTGGTGAAGGAGCACAAAGCCTCAGGTTTTACACTGTTTGGAGGATTTTACTGGATATAGATTAAGGATTGGTTAAGGATGGACATGTTCAACATAATACAAGCCTTGATTTAAAAAAAAACAAAAACAAATACTTTGTCAGAGCTCCTAGATTTTGGAGGGAGCTCTGTTTTTTGGTTTGTTCAACTTTTTTGACACTCTTAATTCTGATACTGGAATGACCTTTCTGAAAATGATTGGATAGTTGAAAAGACTGTTGTCTTGTGATATTTTGGATTACATTGAAACAGCAGAGTTTATTATCATTCTTGACTCATCTGTTCATTGACATTTTGGgtaagttctt...3’

The first nucleotide in RNASeq transcript RNASEQT00000017723 (annotated in Zv9 assembly only), RefSeq transcript XM_001921276 and 5’-RACE product are shadowed in colour code. Positions of forward primer used to clone cDNA is underlined.

**B) exon 2L (TSS2)** is 5’ extension of exon 2

5’...ttggctaatgGATCATATTTACTGAAATGTATTCATATTGCAGGTCACTCTGACAAACAGAGACATTCCTTCCTCCAAATGAAGgtatgtaaat...3’

The first nucleotide in RNASeq transcript R4.

**C) exon 7 (TSS4)**

5’...tttgcagGCTTCCAACCCACTAGATGTGGTGTCAAGACCAGGATCAGTGCCACACCAACACTTGCAGAAAGATTCAAGGtgaagtt...3’

The first nucleotide in RNASeq transcripts R6-R8.

**D) exon 11L (TSS5)** is 5’ extension of exon 11

5’...aatgagAGGAGTTGTAAAAAGGGGGGCATGAGCAAAAACTTTAAAAAAAAAACCTTGTGGTGACAAATCATTAAAACCATGTGAAATGAGCAGAACTAAGCTAAAATGAATAAATGAATGAATGAATGAAGAGTGTTCAGTAATTAATGAAAACATAAATGTTATAAAGACTATATAGCAGTATCAGGACTGTCCTCATCCAGTATCAACATTTCTCTTGTCAGGTCCCACACACCCTTCAGTTTCACCCTACGGATCCTTACAGCATACTCCAAGCAATCTCCAGTCTGGTCTTGTCCCCCATCATGgtaagga...3’

The first nucleotide in RNASeq transcripts R9 and R10.

Figure S5. Multiple transcription start sites (TSSs) in *auts2b* gene locus.

The first nucleotides (TSSs) annotated in RNASeq, RefSeq, Ensembl and 5’-RACE transcripts are shadowed in green, yellow and red colours, respectively. ID numbers of RNASeq transcripts are provided in Table S3. Exonic and intronic sequences are shown in upper and lower cases, respectively. Constitutive exons are highlighted in grey colour.
