# Supplementary material for: Transcriptional Complexity and Distinct Expression Patterns of auts2 Paralogs in Danio rerio
Source: G3 (Bethesda). 2017 Jun 16;7(8):2577–93. doi: 10.1534/g3.117.042622 (PMC5555464; doi:10.1534/g3.117.042622)
Supplement: Supplementary file 6 [file 2577FigureS6.docx]

**A) exon 1A (TSS1 and TSS2)**

5’...cggagtaa**tataataaa**ccagctcacttcctctggcaatcgcagaagtggcggaagtacgtcagtttgcttGACTCGGAGCATTTACACTTCGCAGTGCGATTTCTGACGAGCAAGAAGAGAGAGAAGCGGAGATGTGAATCATCCGCTGTCTCTTTAAAGGCACTTAGCGAAGCTTCACGTATTCCGACAAACACGCCGTCGTCTCTGCCTTCAGCCGCAGCGCGCGCGGGCAGCGTTGAGGAATGGACGCCTGCAACTTACTCTCTTCTGGATAACGAACTCTTTTCTTGCTTGGATATGGTTTGTTTCTGGTGGATTGCGTGCTGAGGAAGGATCTTTGGGGATATATATAAGAGCGTTGACGCGGACCGCGTTGTCTCGGATGGTTTTGTGGCGTCTCTTCACGCTGAAACTTGCTGATAGAATAAGTTCCTATTTTTAAATTGCGCGACACACACGCGGAGGCTTATTTATGCTGTTTCTGACACAGTGGCTCATGGATTGGGTGGAAATTAAAGCTGGGCCGGCGACATGAGTCCAGAGCGCGGTCTGCAGTATGTACACAAGCTGATGTTATTGTATAGCGTTGCAACAAAGTTGCCGTGCGCGTGGAATTCACGCTACTGCAATTAAACAGCCATTTTGCACCTGTTTTAATGAAACAAGGCGTTTCGATCCTGTGACGCTGTGCTTTGGATTATAGCTACACGCCTCAACCTGCATGGATCACATTTGTTTGTTTATCACATTACGGAGCTTAACATCCAAGGTCATACTGTAAATTGTTTACAGGCTTCAGTATCTTATGGGTGTGTTTCACTGATTGTGTGGAATCGATTGGTTTTTACGTGATCGGGTGTCATTTTTTCTTTTGGGTTTTTGCGGGGGGGCGGGGGGAGGACATCGCACCGATTTTAAA**ATG**GAAGGGAAAGTGAAGCAGAGTCGCAGATCCCGTTCGCAGCGGGAGAGGGGACGGAAACGGGAGGCACGCGCCGGGGAAGCCCGCAATCGGAGCCCCTCGTCTGGTTCCGAACGGGAGCGAAGTCCCGGTAAAAACGCTCCGCCACGCTCGACATCCTCGAACAGAACACCGCGACCCCCGCGGAGGAAGAGACGCGAGTCTAGCTCTCAGGAGGAGGATATTATTGATGGATTTGCTATAGCCAGTTTCGTCAGTTTGGACCGTCTCGAGgtaagaacca...3’

TSS1: 5’ ends from 1 to 5; TSS2: 5’ ends from 6 to 7. Predicted TATA-box is highlighted in bold. Position of forward primer used to clone cDNA is underlined. Translation start codon is shown in bold blue.

B) exon 2L (TSS3) is 5’ extension of exon 2

5’...ttttgcaGTAGCTCAGCTCATACTATTGCATTTATCACCTCTAACCTCTCGTTTTTTCTTGTCTCGTTTTCCTCTTTCAGAACAAGAATGTTTCCGTTAAACTGGAAAAAAAGAAATGGGAGGAGATTGTGGTGAAACGACAGAAAGAGACCAAGGAGAGCGTGACCCCTGACATAGACGAGACCGACTTTGCCAATCTGGCCACCAGCATGGGCCGAGAGCACGATCGGGTGAAAGATCGACTGCTGAAGAACACGTACTCCAAGAAGAACAAACGAgtgagtctgt...3’

The first nucleotides in RNASeq transcripts R3 (G), R1 (C) and R2 (A).

C) exon 1B (TSS4)

5’...attgaaagtcGTCCATAAGACATATATAATTGTCTGCTGGAGTGAGATTAAAAAAAAAAAAAGATCTGGTCTGAATTAAACTCCGCCTCTCATACATCGATTGCTTTGGTCTTGTCCTGTGCATTTTTTTAACACACCCAGCGGAGTGGCCGCTTGAAGCGCACTTCAGCGCGCGCTCACGGAACACTCGCTCCCGTATGTGCCTGGAGAGCCTCGCGCAGCGGAGGCGCCGGACGCGCTCAATTCGTGTGGGGACTTTTAAAACCTTCCAGATACGTCGAAAACGACTGGATTCGATCACTTGCTCGAGATAAACGAGATTACCAAGCGCTCAAACCCATGGAATGTTGTTCTGCGGTGTTTTGGCTGAGAAACCGAGCGGTACTTCTGCTGGGAATCTATTGTTAATGATCTGTCATAGGAGAGCTTCCACGACTTGGGAATCATATCTATCGCCTCACCTTACCTTGACTAAAAACCTGATTCTATTCACCTGCACGGGGAATGTATGACTCATTGCTGAACAGAAATCAGTCTGTCTGATCTGGCGTTGAAAATATCCTCCGCTTCTGACAGCTTTCGCCACCGCAGTCGTTAAAAACGCTTCTCTCAAGATTTATTGTTGGGACAGGTTCGTATTTCTTGTCCGCCTTCAGTCGGTAGCGAGCACGGCGAGGGGAAAAAGAGAGAGGAAAAACCTGCCGTTGCTTTCATAAGGCATATACTGGATGTAACGGAATTGCATTTTTATTTGTCACCTTTGGCCAAATCGGTGTTTTTCTCCGATGGACGCGAGTCTGGTGTAGAATTCGgtaaggatat...3’

The first nucleotide in RNASeq transcript RNASEQT00000006457. Transcript was annotated in Zv9 assembly only.

D) exon 6L (TSS5) is 5’ extension of exon 6

5’...gatggatggaTTATTGAGAAGGAAGGATAGATGGAGGTTTAGTAGAAGTGAGTACACATGTAACAAGGTGAATGAATAAAGGCAACAGATGGATGAATAGATAGTGGATGAATGGATCTACAGTGGATGGATGGATGGAACCACAAACTTAACACAACTTCTCTCTCTCTCTTTCTGAATTCAGTCCATAGTGTGCGGTATGTAGTGATAGACTGCTCTGTTCATAGAAACAGCTGGAATGTGTGCACTGGCATTCTTTGCTACCCCTGTGTATAGTGTGTGAAGAATTTAAGAGTTCTTTCTCTTCGTTAAGAGCCCCATGTCCTGTCTGTCAATGACTGTGTGTTTTTGTAAGAGCATGGCATGTTTTCTTTTTCTCGTCGCTTGCCAGAAACTACACGCTCTGGGTACATGGAGAGGAAGGAAAAGCACACTCATGTTTAGTCTGCATAGCACTTAAGTCACTTTGTCGGAAGATTACTTTCCCCCCATTCTAATCTGCGGATAGGATTTGTGATGTCACCATCCATCACTGCGCTTTTGTCGAATCATTAAGTGTAGGGATGTGGAAAATTGCACAAGCTGGCTCTACTTTTGCACAATGTTTTACTGTATTGTCTATGTTGGCTTTGTTTGTTTGTAAGTAGTTGTTGGTTATAGGGTTTTGTGGCCCAATGGCGCTGTGTTTACCCTCAAAAAAGCTTCATTTCTTGAACATGGTCTAACTGTGTTTCTCTTCTGTTTTGCAGGCATCCGATGTAGGATCAGAGAAGCTTTTCTCGCCTACTGCACCTAAAGgtaagagcat...3’

The first nucleotide in RNASeq transcripts R7.

E) exon 7L (TSS6) is 5’ extension of exon 7

5’..ttaaaaaATTAACTAATTCATTCATTCATTTTCCTTTAGCTAAGTCCCTTATTGATCAGGGGTCGCCAGAGCAGAATGAACCACCAATTAATCCAGCATATGTCCAGCTGCACCTTGTACTGGGAAAAATAGTAACTAATTAAAACTGTAAATGTATAAAATAAATGTAGATCCATATTTTAAACTCTTGTCATGTCTCTCTTTACTCTCTAGTTACGCCAACGAATGAGAGTCTCGGGACCAAAACTTCCAGCTCGACCAAAGTGTCTGGCCTTCAGCGTAGCCAGGAACAGAGTAACGACGTTTCGTTTGTCCCGCCCATCTCCAGCCCGACTCCTGCGTCGCCCCCTACGGGGTCTCCTGCCCCTGCTGCGGCGGCAGCCCCCCCGCCTAGACCCCATCCGACCTCCCCAAGCCCTCTCTCTATCAAGAAGGAGTCTTTGCCCCCTATTCCCACTCCTCCCCTGCTGAGGAACCTATCGCATTCACTGGAGCACAGACTTCCGCCCCCATTGCACCACAGCCGGCCAATCAGCAGTCATCATCATCATCCTTTGCCATACAGCAGTCTTCACGACATCAGgttagtaa...3’

The first nucleotide in RNASeq transcripts R8 and R9.

F) exon 8L (TSS7) is 5’ extension of exon 8

5’..atgtaaaGTATAATCTTATAACTACATTTTGTCGTTTGTAACAAAATCTTGTTTTTTCTTTGTAACCACAGCGCCAACCCTTCAACTCTTCCTCCTAAACACCACCTGCCTCACTCCGCTCATCACCTCAGTGGTCATCTATCATCTCCTGCTCCTCCTCTTCCTCTCTCCATCACCGGACTGCCCTCCTCCCACTACTCCCTTCACTCGCCTTCTCACCTCTCTAGCCATCCGGCCATGTTTGCCACCCCTGCTACACTGCCTCCTCCGCCCACCCTACCGACCAACAGCCTGGTGGTGCCAGGACACCCTGCAGGACCACCTTATCCAGgtaaaaaa...3’

The first nucleotide in RNASeq transcripts R10 and R11.

G) exon 10L (TSS8) is 5’ extension of exon 10 and exon 11L (TSS9) is 5’ extension of exon 11

5’...aagccctctcATGTTTTTGCTCACAGATGCCTTTGATTTTGTTGATATGTTTTTAATTTGCGCTCTTTCTCTCTTTGTGTTTCTCTTTATAGTTCGACAAATACGCCCCGAAGCTGGACAATCCGTTCTTGAGACATTCAAATgtaagtgtcgacttattttgactgcgtaatcttgttttgactgaatatgaaatggattatgtcaagttttagagacatatttgtaaacttcttgcATGTTTTTAAGACTTGGTTTGATGTTGTTTGCAAAGCAAGATAGACAAGCTGTAGGTCAAAGAGATGACAAACGTCAGCAAGCTTTACATTCATTTTGTTTTGTTTTTTCTTTAGTTTTTTCCCTCCTACCCGCCAACAATGCCAGGAATGCCTCCGTTGCTTCCACATTCAGGGCCCTTCAGTTCACTGCAAGGTGCCTTCCAGCCAAAG...3’

The first nucleotides in RNASeq transcripts R18 (A, TSS8) and R20 (A, TSS9).

H) exon 15L (TSS10) is 5’ extension of exon 15

5’...ttaaagttgcACTGAAAAAAGTCCCTTTGAATTAAATGTTATGTGTGTCCCTCAAAAGCAGATGCACTTGGACCCACATAAACTGGACATGAATGGAAAACTTGATCTGTTCAGCCGTCCTCCTGCTCCAGGTGTATTCCCTGGGTTCCCCTACCCTCATGATCTGGCCAGGCCCCTCTTCTCATCAACAG...3’

The first nucleotides in RNASeq transcripts R21 and R22.

I) exon 16L (TSS11) is 5’ extension of exon 16

5’..gaatctgATAAACGTTTGCAAATCTTTAAAATAAGTAATGAAATTACAGCTGGCTTCTTTGTGTTCCTTCTTGACCTGTGTCATTTGCTTAGTTTTTATTCTCGCACTCTGATTCCTAGCTGTCAATCAGATCACTCTCTTTCACTGACAGCCTGATTTTACATCCTGAAAAATAAAAACTCCCTTCAACATTAGCCCGATGAGAGATGATGTGTAGAAACCACCAAACAAACTAGCCTGATCGATGCTCACTGATAACTAGGTCCACACGGAATCTGCACGCGCAGAATTCAGCAGATTTTCTGCAGATTTTCTCTGCCATTTTCAGCCACCTGTAAAAGTGGTTAAATACATTTCAGACTTGGTTTATACCTTTATCTGCCATCCTCCAGGTGCATCTTAATAGCTGCTGTAAATAGGGCCTCACAGCCAATCTGATCACCCCAAAACTTCTGCATGTACAGTAGGCCTATATAGAAGCGTCTAGTGGTTTGGTTATGATTTAAAACTTTGTCTACAATCACTTTAACTATATGATGATTTTGTCATTTTTAATAGGATCAGGACATCCAGCTGCTTCACCCTACGGACCATCTCCTCACCATGCTGGCTTCCTGCCTCCTAGCCATTTGGCAGGTAAGTgtaagtaaa...3’

The first nucleotides in RNASeq transcript R23.

J) exon 17L (TSS12) is 5’ extension of exon 17

5’...gtgacgcaaaAACAAAGCTGGCTTGTAAGAGAAAAGCCGGTTGGAATGGTCGGGCACCTCCGGCCCCGCGTCGCTAATCCCTGTTTGGCATTTTGCAGATCCATTCAGTCGCTCCAGTACCTTTGGCGGCCTCAGCAACCTTTCAAGCAGTGCCTTCGGTGGATTAGGCAACCCAGCCCTCG...3’

The first nucleotides in RNASeq transcripts R24.

K) exon 18L (TSS13) is 5’ extension of exon 18

5’..actggacTGTGATCTGATAAACTGATATTATGTTTCTTGATATTTCAGGGGCCAACAGTGTGTTTGGACCAAAAGAGGGTCCAGGACTGCCGGGACTCAGCAGTCCTCATCATGACACCTGGAATCGGCTGCACCGGACACCACCATCCTTCCCGACCCCTCCACAGTGGCCCAAATCTGTAGATGCAGAGAGAAGCAGCTCAGCAAACAGCCACGACAGAGAGAGGGACCGGGAGCGGGAGAAAGAAAAAGAACGGGAGAGAGAAAAAAGGGACTCTTCAGTTGGGAAAGAAGAGAAGGATAAAGACAGgtttgtg...3’

The first nucleotides in RNASeq transcript R25.

Figure S6. Multiple transcription start sites (TSSs) in *fbrsl1* gene locus.

The first nucleotides in RNASeq transcripts and 5’-RACE products are shadowed in green and red colours, respectively. ID numbers of RNASeq transcripts are provided in Table S4. Exonic and intronic sequences are shown in upper and lower cases, respectively. Constitutive exons are highlighted in grey colour. Only partial sequences of exons 11, 15 and 17 are shown.
