# Supplementary material for: Transcriptional Complexity and Distinct Expression Patterns of auts2 Paralogs in Danio rerio
Source: G3 (Bethesda). 2017 Jun 16;7(8):2577–93. doi: 10.1534/g3.117.042622 (PMC5555464; doi:10.1534/g3.117.042622)
Supplement: Supplementary file 7 [file 2577FigureS7.pptx]

## Slide 1
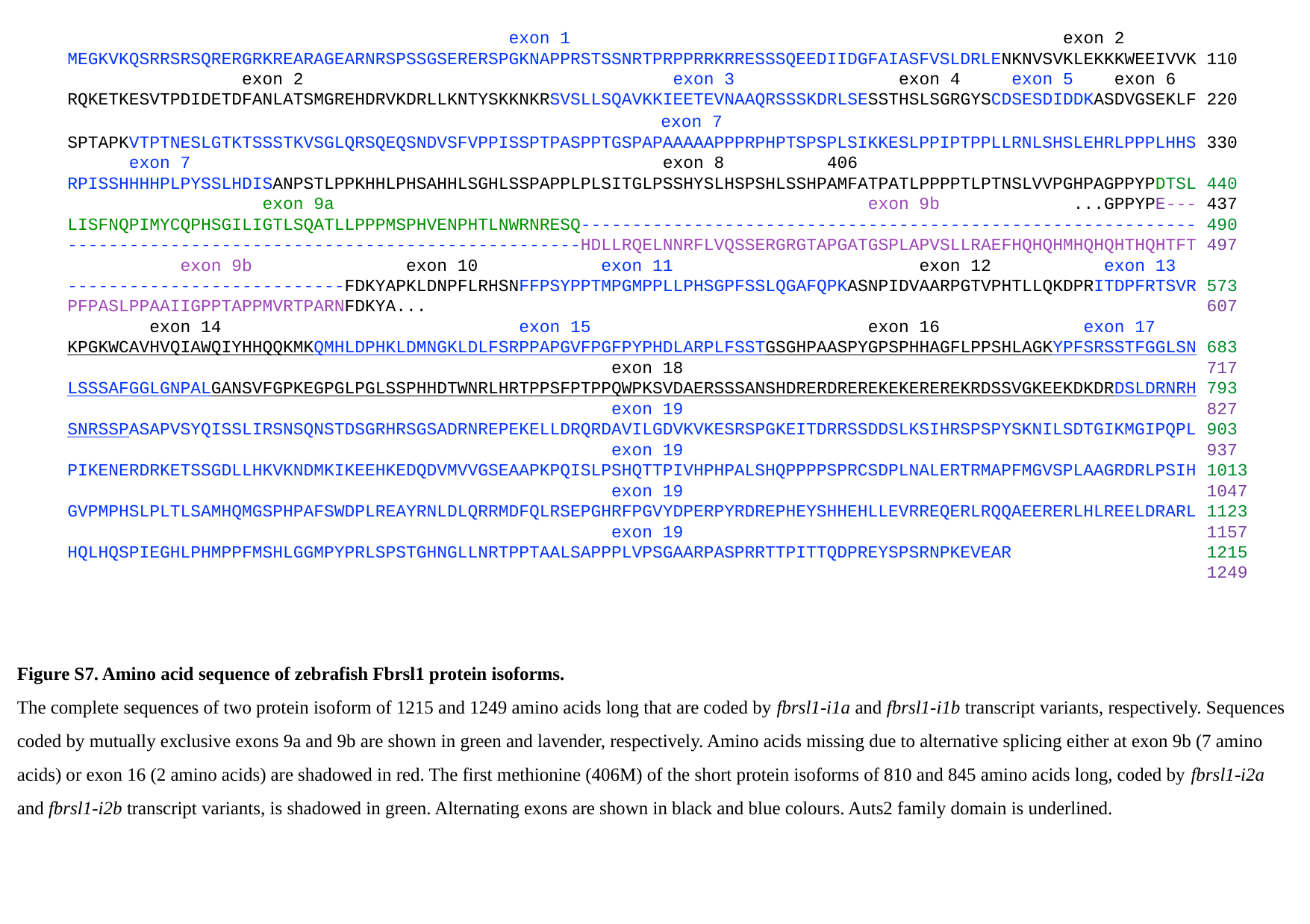

exon 1 exon 2
MEGKVKQSRRSRSQRERGRKREARAGEARNRSPSSGSERERSPGKNAPPRSTSSNRTPRPPRRKRRESSSQEEDIIDGFAIASFVSLDRLENKNVSVKLEKKKWEEIVVK 110
 exon 2 exon 3 exon 4 exon 5 exon 6
RQKETKESVTPDIDETDFANLATSMGREHDRVKDRLLKNTYSKKNKRSVSLLSQAVKKIEETEVNAAQRSSSKDRLSESSTHSLSGRGYSCDSESDIDDKASDVGSEKLF 220
 exon 7
SPTAPKVTPTNESLGTKTSSSTKVSGLQRSQEQSNDVSFVPPISSPTPASPPTGSPAPAAAAAPPPRPHPTSPSPLSIKKESLPPIPTPPLLRNLSHSLEHRLPPPLHHS 330
 exon 7 exon 8 406
RPISSHHHHPLPYSSLHDISANPSTLPPKHHLPHSAHHLSGHLSSPAPPLPLSITGLPSSHYSLHSPSHLSSHPAMFATPATLPPPPTLPTNSLVVPGHPAGPPYPDTSL 440
 exon 9a exon 9b ...GPPYPE--- 437
LISFNQPIMYCQPHSGILIGTLSQATLLPPPMSPHVENPHTLNWRNRESQ------------------------------------------------------------ 490
--------------------------------------------------HDLLRQELNNRFLVQSSERGRGTAPGATGSPLAPVSLLRAEFHQHQHMHQHQHTHQHTFT 497
 exon 9b exon 10 exon 11 exon 12 exon 13
---------------------------FDKYAPKLDNPFLRHSNFFPSYPPTMPGMPPLLPHSGPFSSLQGAFQPKASNPIDVAARPGTVPHTLLQKDPRITDPFRTSVR 573
PFPASLPPAAIIGPPTAPPMVRTPARNFDKYA... 607
 exon 14 exon 15 exon 16 exon 17
KPGKWCAVHVQIAWQIYHHQQKMKQMHLDPHKLDMNGKLDLFSRPPAPGVFPGFPYPHDLARPLFSSTGSGHPAASPYGPSPHHAGFLPPSHLAGKYPFSRSSTFGGLSN 683
 exon 18 717
LSSSAFGGLGNPALGANSVFGPKEGPGLPGLSSPHHDTWNRLHRTPPSFPTPPQWPKSVDAERSSSANSHDRERDREREKEKEREREKRDSSVGKEEKDKDRDSLDRNRH 793
 exon 19 827
SNRSSPASAPVSYQISSLIRSNSQNSTDSGRHRSGSADRNREPEKELLDRQRDAVILGDVKVKESRSPGKEITDRRSSDDSLKSIHRSPSPYSKNILSDTGIKMGIPQPL 903
 exon 19 937
PIKENERDRKETSSGDLLHKVKNDMKIKEEHKEDQDVMVVGSEAAPKPQISLPSHQTTPIVHPHPALSHQPPPPSPRCSDPLNALERTRMAPFMGVSPLAAGRDRLPSIH 1013
 exon 19 1047
GVPMPHSLPLTLSAMHQMGSPHPAFSWDPLREAYRNLDLQRRMDFQLRSEPGHRFPGVYDPERPYRDREPHEYSHHEHLLEVRREQERLRQQAEERERLHLREELDRARL 1123
 exon 19 1157
HQLHQSPIEGHLPHMPPFMSHLGGMPYPRLSPSTGHNGLLNRTPPTAALSAPPPLVPSGAARPASPRRTTPITTQDPREYSPSRNPKEVEAR 1215
 1249
Figure S7. Amino acid sequence of zebrafish Fbrsl1 protein isoforms.
The complete sequences of two protein isoform of 1215 and 1249 amino acids long that are coded by fbrsl1-i1a and fbrsl1-i1b transcript variants, respectively. Sequences coded by mutually exclusive exons 9a and 9b are shown in green and lavender, respectively. Amino acids missing due to alternative splicing either at exon 9b (7 amino acids) or exon 16 (2 amino acids) are shadowed in red. The first methionine (406M) of the short protein isoforms of 810 and 845 amino acids long, coded by fbrsl1-i2a and fbrsl1-i2b transcript variants, is shadowed in green. Alternating exons are shown in black and blue colours. Auts2 family domain is underlined.
