# Supplementary material for: Transcriptional Complexity and Distinct Expression Patterns of auts2 Paralogs in Danio rerio
Source: G3 (Bethesda). 2017 Jun 16;7(8):2577–93. doi: 10.1534/g3.117.042622 (PMC5555464; doi:10.1534/g3.117.042622)
Supplement: Supplementary file 9 [file 2577FigureS9.docx]

**A)** **exon 1** **(TSS1)**

5’...tagcaagaacGACTGAATCTGACCCAGTATTTGTAGATTTACCAGTATCGTAGGCCCCTCAGTGGCAGTAGATGGCGGTACTGTTGTCGTATTCAGTACTGACTGTCTTTAGTGAATCAACAGAGGAAGAAAACCCGACAGAAGCACACAATGCAGTTTGACTGCTACTGGTTATCCACCCAGGCTTCGTGTATGCCCCAGCTTTCGAAATAAAATGAAGAAATCCATTTTTGACGATCGGGACGGAAAGCAAGCTTTCATAGGTAGATTTTCACGTTACGCTTGAGAAAAACGCTGCTCCAGCGGCGGACACCGGCGAAATCTCCAGCTTGACCGCACACAGTGGATCTCACAGCGGGCCGCGGAAGGAGGAGCCCAGCCTGCGCTGACGGGGCTGCGCTCAACATCCTTATCGGTGCAACTCCAGCCCGAGGCTTTGGGGCCTGCTGTATATTATTATCGTTTTAAATGGAGGTTCGAACGAAAGCAGAAGCCAGAGGAAAATGGCCCTCTATACAGCGCGAGTGgtgggtgtga...3’

The first nucleotide in RNASeq transcripts R1-R4, RefSeq transcript XM_003199613.3 and 5’-RACE product are shadowed in colour code. Position of forward primer used to clone cDNA is underlined.

**B) exon 2L (TSS2)** is 5’ extension of exon 2

5’...gattaaaaGCCTCACTGCGCCACTGATGGTTAACTTTGTTTCCCTTTTCACAGACGAAAGTGACAATGTCTTGACAGAAAGACAGTAAGTGGAATATCTGGAATCTTTCATAACTGCTGTGTTGTTGGATAAAACAGGCCCGGTGATGCCCAGACATGACTATACTCAGACTGCAGTGTGTTCACACCATACAGCCTCCGCTGGACTCCATTAGATTATAAGACAGGAACCCAAAGACACATTTCCATCCGAGGTCCACTGCACTGCTTCTGACGGCGTTGTTCTTCATTCTGTAACAGCTGTAAAATCTCTCAGGCCGGACCGGGGTGTGTTTTAATTTAGTGCCTTCGTACGGACGTTGAC**ATG**GACGGTCCTAGTCGGAGCGGAGGCTTCAGGCAGAGCCGTCGCTCCCGATCGCAGCGTGACCGAGAGCGGCGACGGAGGAGAGCGGACCTCACTGAGCACAGGCCCTCGTCGCCATCCTCGGCCTCGGACCAGGAACTTTGCCGAGGAGACTCTCTGCTCCGTGCCGGCGGAGGAGAATGTAGACCCGGCTTCCCCGGGACCAGACACCGGCCTCCGCGGCGGAGGAAGAGAGAGTCGGTGTCTTGCGAGGAGGACATCATTGATGGATTCGCCATAGCCAGCTTCATAAGCCTGGAGGCCTTGGAGgtgtgtgt...3’

The first nucleotide in RNASeq transcript R5. Translation start codon is highlighted in blue color. Position of 5’-RACE primer is underlined.

**C) exon 18L (TSS3)** is 5’ extension of exon 18

5’...atattcatATGCTTGTAGCTGCCAGTGTTTTTGGGCATAAGACTGAGCCCTCTGCAAGTGCTGTTGGTGGACTGGG...3’

The first nucleotide in RNASeq transcripts R6. Only partial sequence of exon 18 is shown.

Figure S9. Multiple transcription start sites (TSSs) in *fbrs* gene locus.

The first nucleotides (TSSs) annotated in RNASeq, RefSeq, EST and 5’-RACE transcripts are shadowed in green, yellow and red colours, respectively. ID numbers of RNASeq transcripts are provided in Table S5. Exonic and intronic sequences are shown in upper and lower cases, respectively. Constitutive exons are highlighted in grey colour.
